# Comparative analysis of mitochondrial and chloroplast genomes of *Dracaena cambodiana* from contrasting island habitats

**DOI:** 10.3389/fpls.2025.1620721

**Published:** 2025-06-25

**Authors:** Heng Liang, Xiuxiu Sun, Huasha Qi, Jiali Chen, Yidan Wang, Chunmei Wang, Moyang Liu, Tengfei Xia, Shiling Feng, Cheng Chen, Daojun Zheng

**Affiliations:** ^1^ Institute of Tropical Horticulture Research, Hainan Academy of Agricultural Sciences, Haikou, China; ^2^ National Germplasm Resource Chengmai Observation and Experiment Station, Hainan Academy of Agricultural Sciences, Chengmaia, China; ^3^ Sanya Institute, Hainan Academy of Agricultural Sciences, Sanya, China; ^4^ Key Laboratory of Tropic Special Economic Plant Innovation and Utilization, Hainan Academy of Agricultural Sciences, Haikou, China; ^5^ School of Life Sciences, Technical University of Munich, Freising, Germany; ^6^ School of Agriculture and Biology, Shanghai Jiao Tong University, Shanghai, China; ^7^ College of Life Science, Sichuan Agricultural University, Ya’an, Sichuan, China

**Keywords:** organelle genome, *D. cambodiana*, comparative genomics, adaptive evolution, genomic resources

## Abstract

**Introduction:**

*Dracaena cambodiana*, a vulnerable species widely distributed in tropical and subtropical areas, has been recognized as a model plant for studying island conservation biology due to its fragmented habitat, slow growth, and ecological sensitivity. However, its organelle genome evolution and population divergence across different island environments remain poorly understood.

**Method:**

In this study, we *de novo* assembled and annotated the complete chloroplast (cp) and mitochondrial (mt) genomes of two geographically distinct individuals of *D. cambodiana* from Hainan Island, China: a coastal area (SY) and a mountainous forest area (DF).

**Results:**

Both genomes showed conserved circular structures, but comparative analyses revealed key differences. The chloroplast genomes exhibited intergenic hotspot regions such as *trnC-GCA–petN*, *trnL-UAA–trnF-GAA*, and *psaI–ycf4*, which may serve as potential markers for taxonomy, population genetics, phylogeography and conservation biology of *D. cambodiana*. In the mitochondrial genomes, three genes (*nad1*, *nad5*, and *rps11*) showed the non-synonymous to synonymous substitution rate ratio (Ka/Ks) >1, indicating potential positive selection linked to environmental stress in the coastal population. Over 580 RNA editing sites were identified in each mitochondrial genome, with minor differences between DF and SY. These results suggest that while organelle genome structures are conserved, subtle molecular variations could potentially be associated with environmental differences between populations, although further investigation is needed to confirm adaptive significance.

**Conclusion:**

This study provides foundational genomic resources for understanding the adaptive evolution of *D. cambodiana* and supports conservation strategies in island ecosystems.

## 1 Introduction

Plant organellar genomes, including mitochondrial (mtDNA) and chloroplast (cpDNA) genomes, are critical tools in conservation biology and evolutionary research due to their maternal inheritance, structural conservation, and sensitivity to environmental pressures (Chen et al., 2014; Wang et al., 2015; Dar et al., 2022; Liang et al., 2025a). They provide unique insights into evolutionary divergence, population genetics, and plant adaptation mechanisms (Gray et al., 1999). Numerous studies have demonstrated that mtDNA mutations are closely associated with plant tolerance to abiotic stresses such as drought, salinity, and extreme temperatures (Yang et al., 2022; Mo et al., 2023). Similarly, structural rearrangements in chloroplast genomes, including inversions and variations in intergenic regions, have been implicated in niche divergence and adaptive radiation in various plant groups (Jaggi et al., 2025). However, despite these advances, organellar genome research focusing explicitly on rare or endangered island plant species remains limited.

Island ecosystems often harbor endemic plants that face unique evolutionary pressures due to geographic isolation and environmental stresses, thus becoming valuable systems for studying conservation and adaptation (Kueffer et al., 2014; Whittaker et al., 2023). Within this context, *Dracaena* species, woody monocots widely distributed across tropical Asia and Africa, have garnered attention due to their ecological significance, ornamental value, and sensitivity to habitat degradation (Maděra et al., 2019; Celiński et al., 2020; Ding et al., 2020).

Among these*, Dracaena cambodiana* is a vulnerable species endemic to fragmented habitats on Hainan Island, China (Li, 2007; Zheng et al., 2012). It represents a suitable model for studying island conservation biology due to its fragmented distribution, ecological sensitivity, and slow growth. These attributes make it an ideal subject to investigate potential local adaptation and genetic differentiation in response to diverse environmental conditions. Despite its recognized conservation significance, studies to date have primarily focused on horticultural practices and propagation methods (Ding et al., 2018). Comprehensive genomic research, particularly comparative analysis of mitochondrial and chloroplast genomes among populations (or individuals) in distinct habitats, remains notably absent.

Recent field investigations identified approximately ten natural populations of *D. cambodiana* in Hainan Island, with each population comprising from 20 to approximately 5,000 mature individuals (Zheng et al., 2012). Although some populations have relatively large numbers of individuals, most exhibit limited regeneration capacity, dominated by older individuals, indicating potential long-term population declines. Populations are restricted to coastal rocky areas and mountainous forests, which experience distinct environmental stresses. Specifically, the coastal population (SY) endures conditions such as frequent drought, high salinity, strong ultraviolet radiation, and nutrient-poor sandy soils. In contrast, populations (or individuals) in mountainous forest habitats (DF) experience more stable and humid environments. A detailed map indicating these distribution areas and sampling sites (SY and DF) is provided in **Supplementary Figure S1**. These contrasting environmental conditions likely impose differential selective pressures that could shape the genetic variation and adaptive evolution in *D. cambodiana*.

While the chloroplast genome of *D. cambodiana* has recently been reported (Zhu et al., 2018), comprehensive comparative studies, especially those involving mitochondrial genomes across populations (or individuals) from ecologically contrasting habitats, remain lacking. Clarifying genomic diversity and adaptive genetic variation within this species is essential for its effective conservation management, especially under conditions of environmental stress.

To address these research gaps, we *de novo* assembled and annotated the complete mitochondrial and chloroplast genomes of two *D. cambodiana* individuals from contrasting habitats. Comparative chloroplast genome analysis with other species was performed to identify intergenic hotspots with potential taxonomic and conservation value. In addition, we conducted a detailed comparative analysis of mitochondrial genome structure, repetitive elements, codon usage, RNA editing, and selection pressure. The results aim to provide new insights into adaptive evolution in island environments and contribute genomic resources for future studies on conservation biology, population genetics, and species management.

## 2 Material and method

### 2.1 Collection of plant material, extraction of DNA, and genome sequencing

Young and healthy leaves of *D. cambodiana* were collected from the coastal area (109°09′5.50′′ E, 18°18′3.08′′ N, altitude=15m) and Mountain areas (the tropical forests) (109°04′11.90′′ E, 18°59′58.00′′ N, altitude=980m) from Hainan island in China (**Supplementary Figure S1**). The leaves were frozen with liquid nitrogen. Total genomic DNA was extracted using a Plant Genomic DNA Kit (Tiangen Biotech Co., Ltd., Beijing, China) according to the manufacturer’s instructions. The DNA was assessed with a NanoDrop 2000 spectrophotometer (Thermo Scientific, USA) and stored at −20°C for future use.

The complete mitochondrial and chloroplast genomes of *D. cambodiana* were obtained from total genomic DNA using a hybrid sequencing approach. Long-read sequencing was performed using the Oxford Nanopore PromethION platform. Libraries were constructed with the SQK-LSK109 kit (Oxford Nanopore Technologies, Cambridge, UK). The Nanopore sequencing generated 1,198,179 (DF) and 2,007,685 (SY) raw reads with average lengths of 9,263 bp (DF) and 6,992 bp (SY). Raw reads were filtered and quality-assessed using NanoFilt and NanoPlot tools from the NanoPack suite (De Coster & Rademakers, 2023). Post-filtering, 1,102,763 (DF) and 1,838,343 (SY) clean reads remained, with average read lengths of 9,251 bp (DF) and 7,011 bp (SY).

Simultaneously, short-read sequencing was conducted using Illumina NovaSeq 6000. Short-read sequencing libraries with an average insert size of 350 bp were prepared using the Nextera XT DNA Library Preparation Kit (Illumina, San Diego, CA, USA). Illumina sequencing yielded 38,159,204 (DF) and 35,777,312 (SY) raw reads. Fastp (v0.20.0, Chen, 2023) was used for quality filtering and trimming, resulting in 32,506,258 (DF) and 33,151,076 (SY) high-quality short reads.

### 2.2 Genome assembly and annotation

For mitochondrial genomes, Minimap2 (v.2.24) was used to align Nanopore reads to our draft assembly of *D. cambodiana* (Li, 2018). The aligned reads were then extracted and assembled *de novo*. Initially, Unicycler was employed, followed by Flye (v.2.9.3) (Kolmogorov et al., 2019). The assembled result was polished using Racon (v.1.4.3) (Vaser et al., 2017). Subsequently, bowtie2 (v.2.5.4) (Langmead and Salzberg, 2012) aligned short reads to the corrected assembly. Unicycler (v.0.5.1) (Wick et al., 2017) was then used for a mixed assembly, and finally, the assembly was refined according to the coverage of the long reads to produce the final result. The annotation of the mitochondrial genome structure involved several steps. First, the genomes were annotated using BlastN (v.2.6.0) and Mitofy. Identified mitochondrial genes were compared against the NCBI database (https://www.blast.ncbi.nlm.nih.gov, accessed on 10 August 2024). tRNA genes were identified with tRNAscan-SE software (v.2.0.12) (http://lowelab.ucsc.edu/tRNAscan-SE/, accessed on 10 August 2024). The newly sequenced mitochondrial genomes were deposited in GenBank under accession numbers PQ279226 (DF) and PQ279227 (SY), the chloroplast genome assemblies were deposited under accession numbers PV477048 (DF) and PV477048 (SY), and genome maps were created using the CGView Server.

GetOrganelle (v.1.6.4, parameters: -R 15 -k 21,45,65,85,105) was employed to assemble the complete chloroplast genome (Jin et al., 2020). Following assembly, the circular cp genomes were annotated using CpGAVAS2 and GeSeq online tools, with reference to the existing chloroplast genome (Wang et al., 2022; Liang et al., 2024). Apollo was then used to correct start codons, stop codons, and intron/exon boundaries (Lewis et al., 2002).

### 2.3 Chloroplast genome structure and comparative analysis

A map of the chloroplast genome structure was created using OGDRAW. The IR region tends to be more conserved than the LSC and SSC regions (Zheng et al., 2020). Differences in fragment length and gene types at the IR boundary reveal a species’ unique evolutionary path. For comparison of the LSC/IR/SSC boundary regions, we included the chloroplast genomes of *D. cochinchinensis* (MN200195) and *D.* sp. (OR601564) obtained from NCBI GenBank. In this study, IRscope (Amiryousefi et al., 2018) was used to analyze and visualize the IR boundary in the two chloroplast genomes sequenced, as well as in two other *Dracaena* species. mVISTA was used to aligment and visual the four chloroplast genomes (Frazer et al., 2004).

### 2.4 Mitochondrial genome structure and comparative analysis

RNA editing sites in the mitochondrial genome of *D. cambodiana* (SY and DF) were identified by referencing plant mitochondrial gene-encoded proteins. The Plant Predictive RNA Editor (PREP) suite (http://prep.unl.edu/) was used for the analysis to reduce redundancy. Codon usage and relative synonymous codon usage (RSCU) for mitochondrial protein-coding genes were analyzed using MEGA software (v7.0).

Repetitive sequences are prominent features influencing genome evolution, heredity, and variation (Jurka et al., 2007). They play essential roles in gene expression, transcriptional regulation, chromosome structure, and physiological metabolism (Shapiro and von Sternberg, 2005). Tandem repeats in the mitochondrial genomes of *D. cambodiana* (SY and DF) were analyzed using Tandem Repeats Finder (v4.0.9) software (Benson, 1999). Simple sequence repeats (SSRs) were identified using MISA (Beier et al., 2017). Homologous sequences between chloroplast and mitochondrial genomes were analyzed using BLASTN software with default parameters, and homologous fragments were visualized by the Circos (Krzywinski et al., 2009). The non-synonymous substitution rate, synonymous substitution rate, and the ratio of Ka/Ks were determined using KaKsCalculator2 (Wang et al., 2010), and R package (ggplot2) plotted boxplots of paired Ka/Ks values). The Conserved protein-coding genes (CDSs) were also extracted by Phylosuite software (v1.2.2) (Xiang et al., 2023). Nucleotide diversity (Pi) values for shared CDSs were calculated using DnaSP v6.12.03 (Rozas et al., 2017).

### 2.5 Phylogenetic tree construction

To determine the phylogenetic position of *D. cambodiana*, we obtained the complete mitochondrial and chloroplast genome sequences of 15 plant species. CDSs shared across species were extracted separately from each organellar genome, and independent phylogenetic trees were constructed based on the mitochondrial and chloroplast CDSs datasets, respectively. Multiple sequence alignment was performed using MAFFT (v7.427) with the –auto option (Katoh and Standley, 2013). Phylogenetic trees were generated using two methods: 1. Maximum Likelihood (ML) analysis conducted with RAxML (v8.2.10) employing the GTRGAMMA model and 1000 bootstrap replicates for branch support (Stamatakis, 2014). 2. Bayesian Inference (BI) analysis was performed with MrBayes (v3.2.7a) (Ronquist et al., 2012) based on the model selected by jModelTest (v2.1.10) (Posada, 2008). The BI analysis was run for 2 million generations with sampling every 100 generations, and convergence was assessed based on average standard deviation of split frequencies (< 0.01) and effective sample size (ESS) > 200. *Crocus sativus* served as the outgroup species. Finally, the GTR+I+G was identified as the optimal model for our dataset by Akaike Information Criterion (AIC) for the datasets.

## 3 Results

### 3.1 General features of the *D. cambodiana* chloroplast and mitochondrial genomes

In general, the average sequencing depth for the chloroplast genomes of the DF and SY populations was approximately 1,498 X and 1,925 X, respectively, with 100% of the genome covered at a depth greater than 400 X. For the mitochondrial genomes, the average read depth reached 395 X (DF) and 285 X (SY), with genome coverage above 100% (**Table 1**). These high coverage values support the reliability and completeness of the *de novo* assemblies.

**Table 1:** The information on chloroplast genomes and mitogenome of DF and SY.

Types	Samples	Avg Depth (×)	genome size	GC content	Gene	mRNA	rRNA	tRNA	Pseudo gene
chloroplast genomes	DF	1,498	155,397	37.6	133	84	8	38	3
	SY	1,925	155,297	37.6	133	84	8	38	3
mitogenome	DF	395	521,845	49.65	64	36	3	24	1
	SY	285	521,897	49.66	64	36	3	24	1

**Figure 1:**
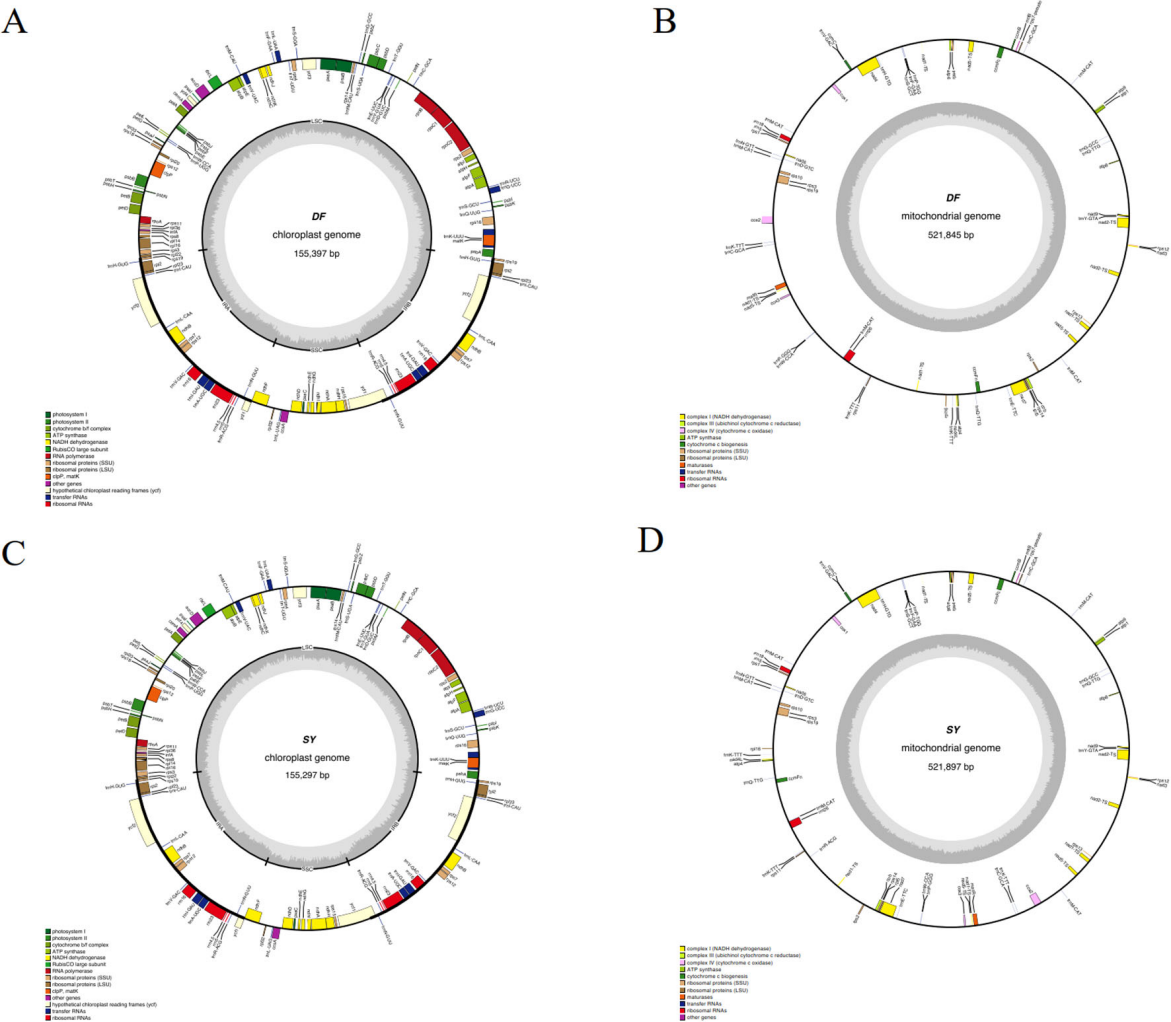
The maps of organelle genomes of *D*. *cambodiana*. **(A)** chloroplast genome of DF; **(B)** mitogenome of DF; **(C)** chloroplast genomes of SY; **(D)** mitogenome of SY.

Based on the read numbers of Illumina, the circular chloroplast genomes of *D. cambodiana* were obtained, which 155,397 bp in DF (from the coastal area) and 155,297 bp in SY (from Mountain areas, **Figures 1A, C**). Both genomes consisted of LSC (large single-copy), SSC (small single-copy), and two IR regions, and contained the same number and types of genes: 84 protein-coding genes, 38 tRNA genes, 8 rRNA genes, and 3 pseudogenes (**Table 1**). The two samples displayed similar gene numbers, GC content, and genome sizes. The genes were categorized into four groups (**Supplementary Table S1**). Among these, 16 genes contained multiple introns, with 3 genes having two introns each. To provide a broader comparative perspective, we also compared our newly assembled chloroplast genomes (DF and SY) with the previously published *D. cambodiana* chloroplast genome (Zhu et al., 2018). The three genomes showed conserved quadripartite structures, similar gene content, and comparable GC content.

The complete mitochondrial genome of DF is 521, 845 bp in size (total), and contains a GC content of 49.65% (**Figure 1B**). In SY, 521,897 bp was discovered, with a GC content of 49.66% (**Figure 1D**). The assembly statistics summary of SY and DF was showed in **Table 1**. The mitochondrial genome of SY and DF were annotated, revealing the same 64 distinct protein-coding genes, 36 mRNA genes, 24 tRNA genes, and three rRNA genes (**Table 1**). Among the 64 protein-coding genes, 24 were identified as core genes and 13 as non-core genes (**Supplementary Table S2**). The core genes included five ATP synthase genes (*atp1, atp4, atp6, atp8*, and *atp9*), nine NADH dehydrogenase genes (*nad1, nad2, nad3, nad4, nad4L, nad5, nad6, nad7*, and *nad9*), four cytochrome c biogenesis genes (*ccmB, ccmC, ccmFc*, and *ccmFn*), three cytochrome c oxidase genes (*cox1, cox2*, and *cox3*), a transport membrane protein gene (*mttB*), a maturase gene (*matR*), and a ubiquinol cytochrome c reductase gene (*cob*). The non-core genes comprised two large ribosomal subunit genes (*rpl16* and *rpl5*), eleven small ribosomal subunit genes (*rps7, rps1, rps10, rps11, rps12, rps13, rps14, rps19*, *rps2, rps3* and *rps4*).

### 3.2 Structure and IR boundary variation of chloroplast genome

**Figure 2:**
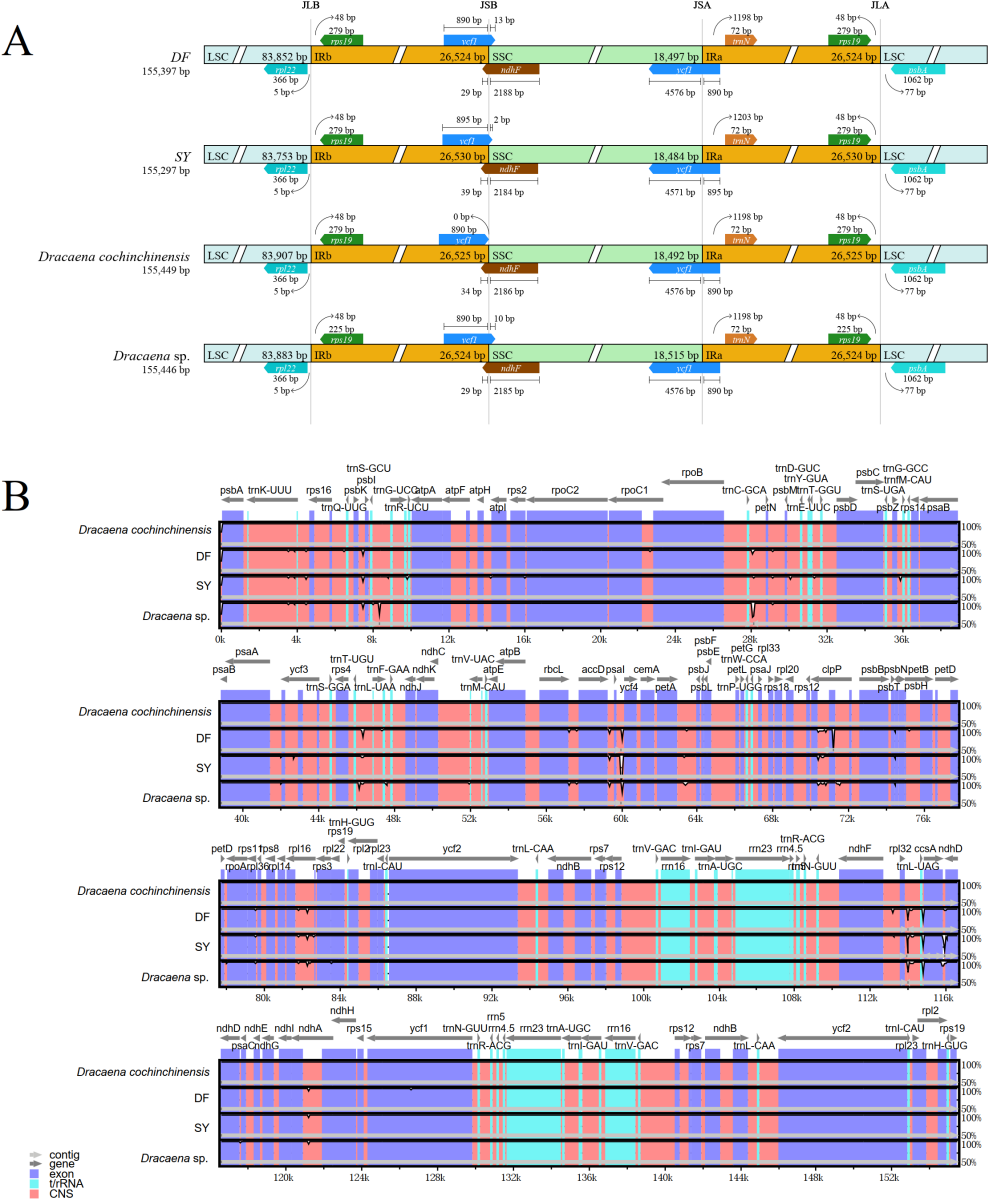
Variations in genome structure. **(A)** Comparison of the boundary between LSC, IR, and SSC regions among four *Dracaena* chloroplast genomes. JLB, the junction between LSC and IRb; JSB, the junction between SSC and IRb; JSA, the junction between SSC and IRa; JLA, the junction between LSC and IRa. **(B)** mVISTA analysis among four *Dracaena* chloroplast genomes.

The expansion and contraction of IR regions are common evolutionary processes in chloroplast genomes, causing variations in genome length, gene copy number, and the formation of pseudogenes. Here, the junctions between the LSC, IR, and SSC regions were analyzed in the chloroplast genomes of DF, SY, and two other previously sequenced *Dracaena* species. The IR region length of these four chloroplast genomes were ranging from 26,524 from 26,530 bp (**Figure 2A**). The results indicated that in *D. cochinchinensis*, a copy of *ycf1* was entirely situated in the IRb region. In contrast, variable lengths of *ycf1* were found embedded in the SSC region of DF (13 bp), SY (2 bp), and *D*. sp. (10 bp). Another copy of *ycf1* was positioned at the junction between the SSC and IRa regions, only SY showed variations in gene length and the extent of expansion. The positioning of *ndhF* and *trnN* was also flexible, particularly for the *ndhF* gene at the IRb and SSC regions. This implies that the chloroplast genome changes that must occur in order to interpret the environment.

The mVISTA showed differences throughout all the chloroplast genomes, with the non-coding regions exhibiting the most variability. (**Figure 2B**). The LSC region showed greater divergence compared to the SSC and IR regions. Additionally, we identified numerous ‘hotspot’ regions, including *trnC-GCA-petN*, *trnL-UAA-trnF-GAA* and *psaI-ycf4*. In SY and DF, we also identified ‘hotspot’ regions, like *psbK-psbI*, *trnC-GCA-petN, trnL-UAA-trnF-GAA* and *clpP.* The significant variations within the shared genes (like *clpP*) and intergenic regions (like *trnL-UAA-trnF-GAA*) may be valuable for future taxonomic and phylogenetic research.

### 3.3 RNA editing, codon usage bias, and repeat sequence analysis in the mitochondrial genome

**Figure 3:**
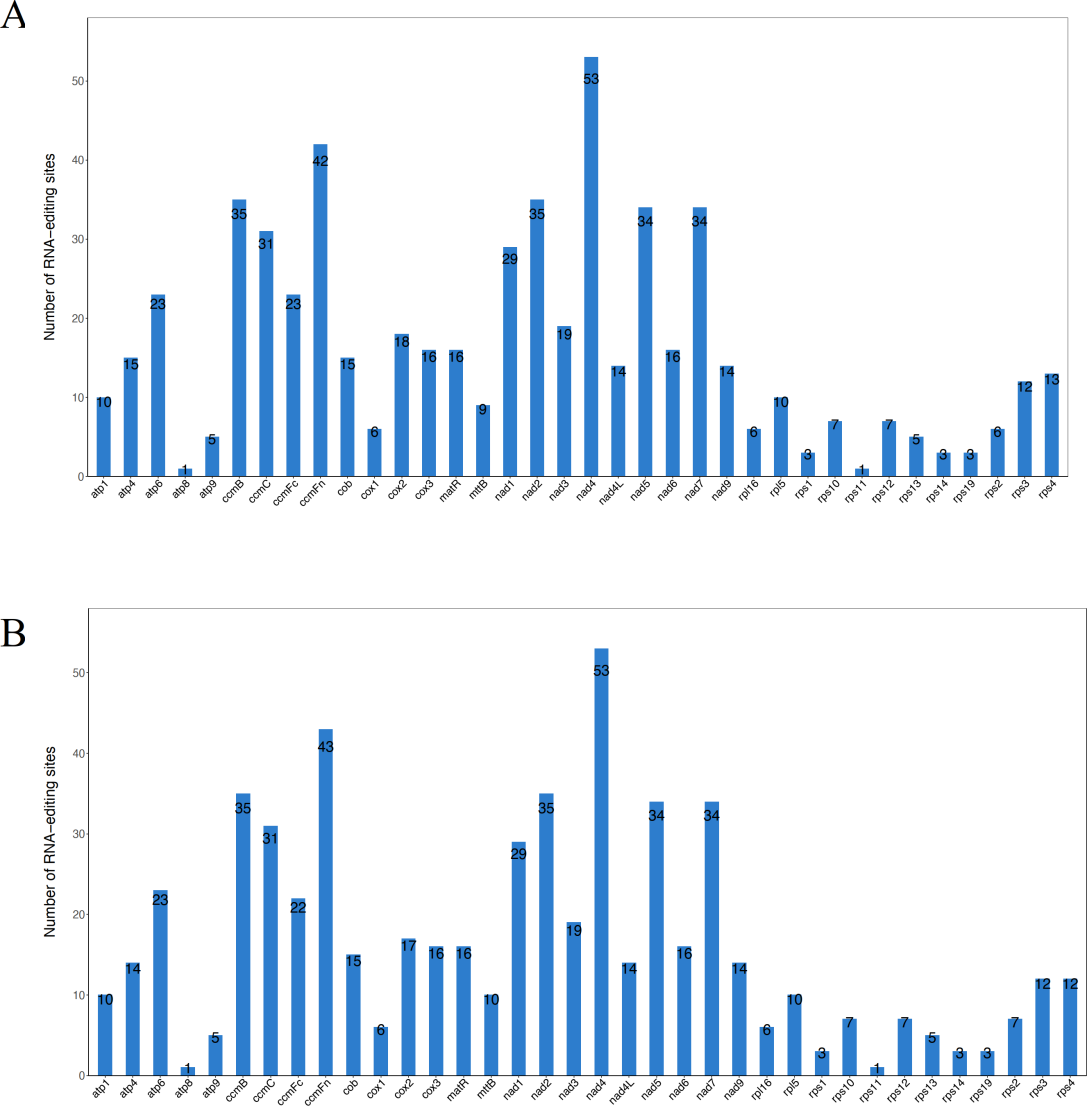
Prediction of RNA editing sites based on the CDSs in **(A)** DF and **(B)** SY.

In eukaryotes, RNA editing refers to the addition, loss, or substitution of bases within the coding region of transcribed RNA (Takenaka et al., 2013; Yablonovitch et al., 2017). This study predicted a total of 589 and 588 RNA editing sites within 36 protein-coding genes of the DF and SY mitogenome, respectively (**Supplementary Tables S3A, B**). These RNA editing sites were unevenly distributed across genes, with numbers ranging from 1 (in *atp8* and *rps11*) to 53 (in *nad4*) in SY and DF (**Figure 3**). We found that the number of RNA editing sites differed among several genes, such as *ccmFc* (23 in DF and 22 in SY), *cox2* (18 in DF and 17 in SY), and *mttB* (9 in DF and 10 in SY). After RNA editing in DF and SY, it was predicted that 42.78% of amino acids would remain unchanged in hydrophobicity, as well as 8.66% would shift from hydrophobic to hydrophilic, and 47.88% would transition from hydrophilic to hydrophobic.

There were 30 distinct codon transfer types, corresponding to 11 amino acid transfer types. The most common codon transfer type was TCA → TTA, occurring at 75 sites. The results also indicated a strong tendency for amino acids generated after codon editing to convert to leucine. All RNA editing sites in the *D. cambodiana* mitogenome were of the C-to-T type.

Codon usage bias is believed to result from a long-term evolutionary balance within the cell, shaped by selective pressures over time (Xu et al., 2024). Firstly, we analyzed the codon composition of the *D. cambodiana* mitogenome. The total number of codons across all coding genes were 10,441 in DF and SY.

**Figure 4:**
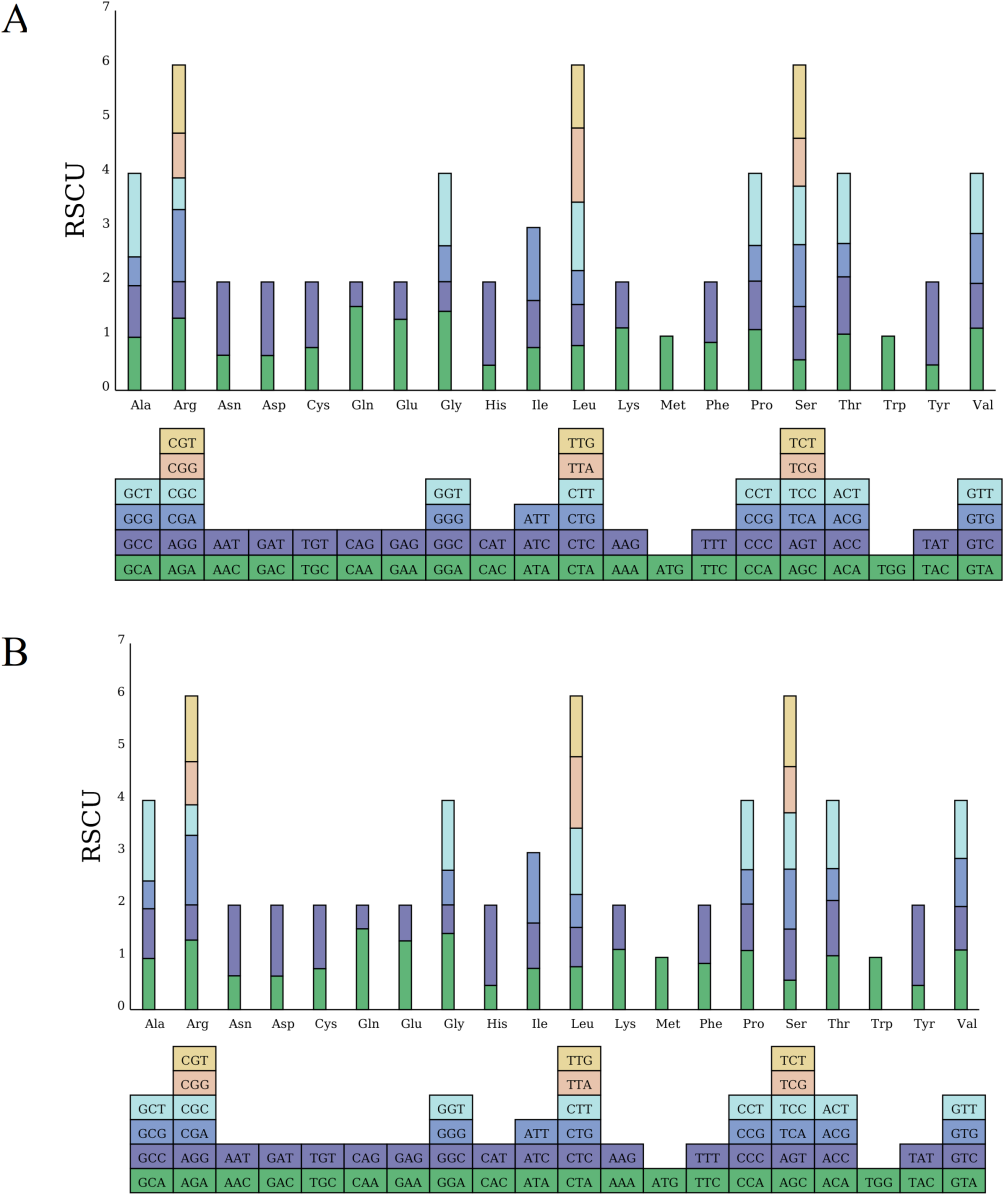
Analysis of RSCU in the mitochondrial genome of **(A)** DF and **(B)** SY. The different amino acids are shown on the x-axis. RSCU values are the number of times a particular codon is observed relative to the number of times that codon would be expected for uniform synonymous codon usage.

Further, the codon usage bias in the *D. cambodiana* mitogenome was assessed using the relative synonymous codon usage (RSCU) metric (**Supplementary Table S4**, **Figure 4**). An RSCU value of 1 indicates unbiased codon usage, while an RSCU value less than 1 signifies that the codon is used less frequently than other synonymous codons, and an RSCU value greater than 1 indicates a higher usage frequency compared to other synonymous codons (Barrington et al., 2023). As shown in **Supplementary Table S4**, 33 codons had RSCU values greater than 1 in the DF and SY, indicating their higher usage compared to other synonymous codons. Notably, 27 of these codons, which end with the A/T base, accounted for 90.00% of the codons with elevated RSCU values. Most of RSCU values were little difference between DF and SY overall. Only the values of UAA (termination codon) showed a different in DF (1.0588) and SY (1.0000).

**Figure 5:**
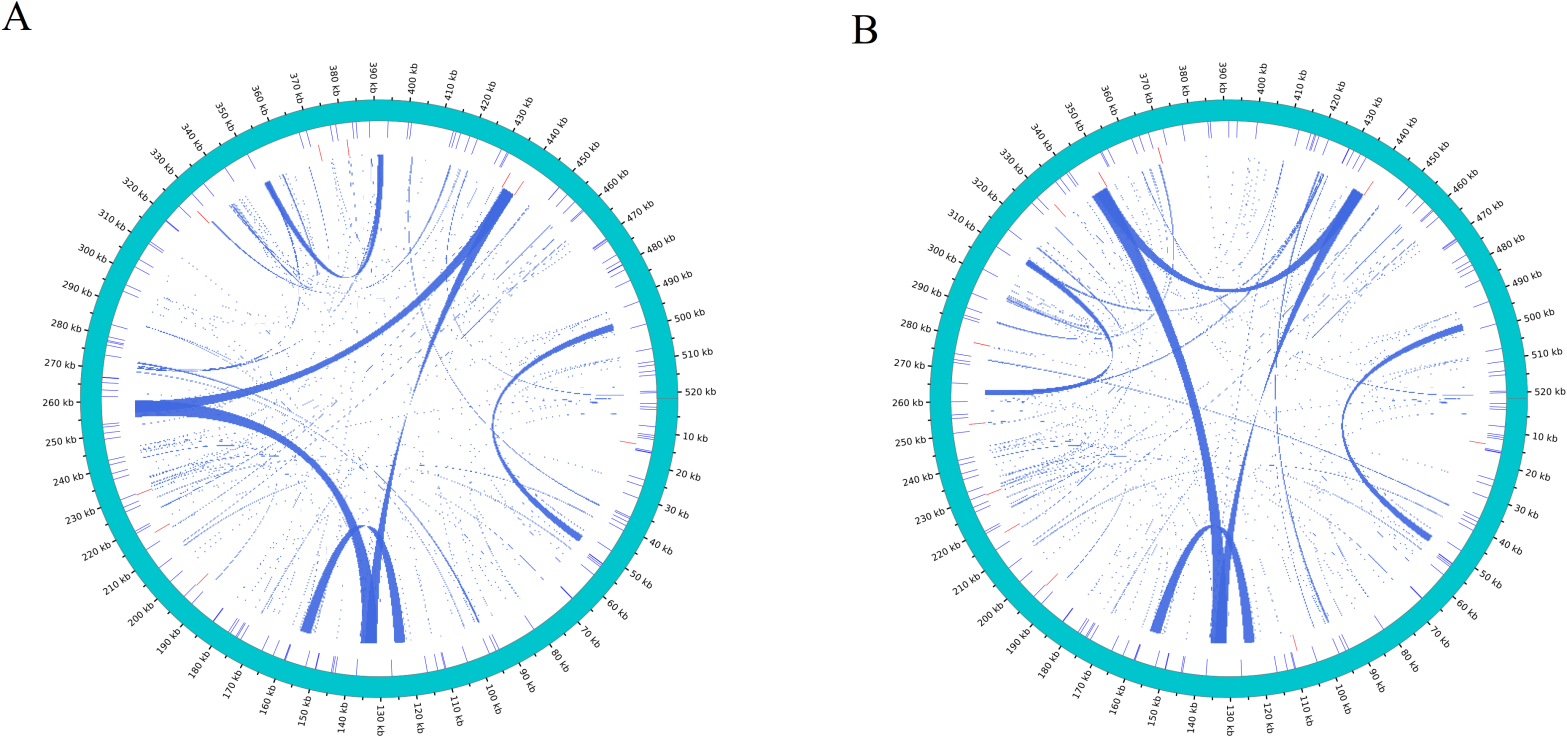
Distribution of repetitive sequences in mitochondrial genome of **(A)** DF and **(B)** SY. The outermost circle represents the mitochondrial genome; the inner circle is SSR (Blue), tandem repeat (red), and dispersed repeat (turquoise).

Repetitive sequences play a vital role in the evolution of plant genomes (Palmer and Herbon, 1988). In the two *D. cambodiana* mitochondrial genome, 139 (DF) and 136 (SY) simple sequence repeats (SSRs) were identified. In DF, monomeric and dimeric SSRs comprised 53.24% of the total (**Figure 5A**, **Supplementary Table S5**). Among the 41 monomeric SSRs, thymine (T) repeats represented 53.6% (22 repeats), with only 2 hexameric SSRs identified in the mitochondrial genome. In SY, monomeric and dimeric SSRs made up 56.62% of the total (**Figure 5B**, **Supplementary Table S5**). Among the 37 monomeric SSRs, thymine (T) repeats accounted for 56.76% (21 repeats). The SSRs identified in DF and SY were 41/36 monomeric, 33/31 dimeric, 18/18 trimeric, 39/40 tetrameric, 6/8 pentameric, and 2/2 hexameric, respectively.

Tandem repeats, also known as satellite DNA, are prevalent in both eukaryotic and prokaryotic genomes (Gelfand et al., 2007). These repeats consist of core units approximately 7 to 200 bases long, repeated multiple times in sequence. We identified 9 (DF) and 11 (SY) tandem repeats in the mitochondrial genome of *D. cambodiana*, with lengths ranging from 17 to 34 bp (**Figure 5**, **Supplementary Table S6**).

Dispersed repeats are a type of repeat sequence that differ from tandem repeats in their organizational form. In DF, 130 pairs of repeats with lengths of 31 bp or more were identified. This included 59 pairs of palindromic repeats and 71 pairs of forward repeats, but no reverse or complementary repeats were found (**Figure 5**, **Supplementary Table S7**). The longest palindromic repeat was 5,612 bp, and the longest forward repeat was 4,076 bp (**Supplementary Table S7**). In SY, 137 pairs of repeats with lengths of 28 bp or more were identified, including 71 pairs of palindromic repeats and 66 pairs of forward repeats. The longest palindromic repeat measured 5,617 bp, while the longest forward repeat was 4,073 bp.

### 3.4 Homologous fragments between mitochondria and chloroplasts

**Figure 6:**
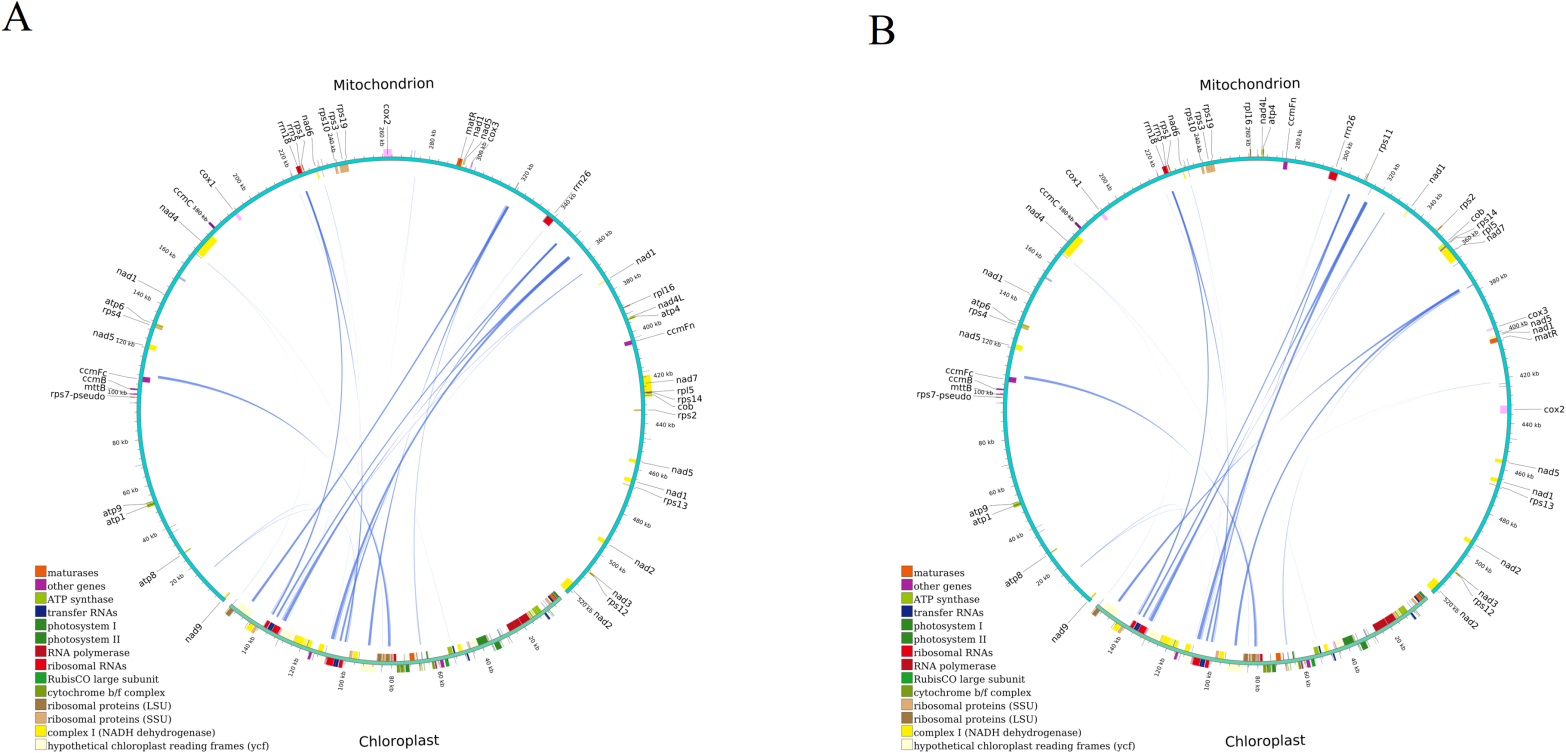
Homologous analysis based on different organelles shows the cyan arc representing. **(A)** DF and **(B)** SY.

Following this, we conducted a homologous fragment analysis between the two *D. cambodiana* mitochondrial and chloroplast genomes using the BLASTn program. In DF, 25 homologous DNA fragments (mitochondrial plastid DNAs, MTPTs) were identified (**Figure 6A**, **Supplementary Table S8A**). Of these, 7 fragments were over 1,000 bp, with the longest 1,527 bp and the shortest 67 bp. The combined length of these fragments was 6,962 bp, comprising 1.33% of the mitochondrial genome and 12,125 bp in chloroplast genome (7.8%). Among the 25 homologous fragments, we annotated 13 complete genes, including 8 genes in chloroplast genome (*rrn4.5; rrn5; trnV-GAC; trnW-CCA; trnP-UGG; trnH-GUG; trnN-GUU; trnM-CAU*) and 5 tRNA genes in mitochondrial genome (*trnW-CCA; trnP-GGG; trnH-GTG; trnN-GTT; trnM-CAT*). Additionally, our findings indicated that three CDSs, *ycf2 rpl14* and *rps8*, have migrated from the chloroplast genome to the mitochondrial genome.

In SY, 26 homologous DNA fragments were found (**Figure 6B**, **Supplementary Table S8B**). Among these, 4 fragments exceeded 1,000 bp, with the longest 1,584 bp and the shortest 67 bp. Their combined length was 6,999 bp, accounting for 1.34% of the mitogenome and 12,234 bp (7.88%) of the chloroplast genome. Annotation of these fragments revealed 15 complete genes, including 9 genes in chloroplast genome (*rrn4.5; rrn5; trnR-ACG; trnV-GAC; trnW-CCA; trnP-UGG; trnH-GUG; trnN-GUU; trnM-CAU*) and 6 tRNA genes in mitochondrial genome (*trnR-ACG; trnW-CCA; trnP-GGG; trnH-GTG; trnN-GTT; trnM-CAT*). Four CDSs *(ycf2; rpl14; rpl16* and *rps8*) were partical migrated from the cp genome to the mt genome in SY (**Supplementary Table S8B**), and most of them lost their integrity during evolution.

### 3.5 Selection analysis in mitochondrial genome

**Figure 7:**
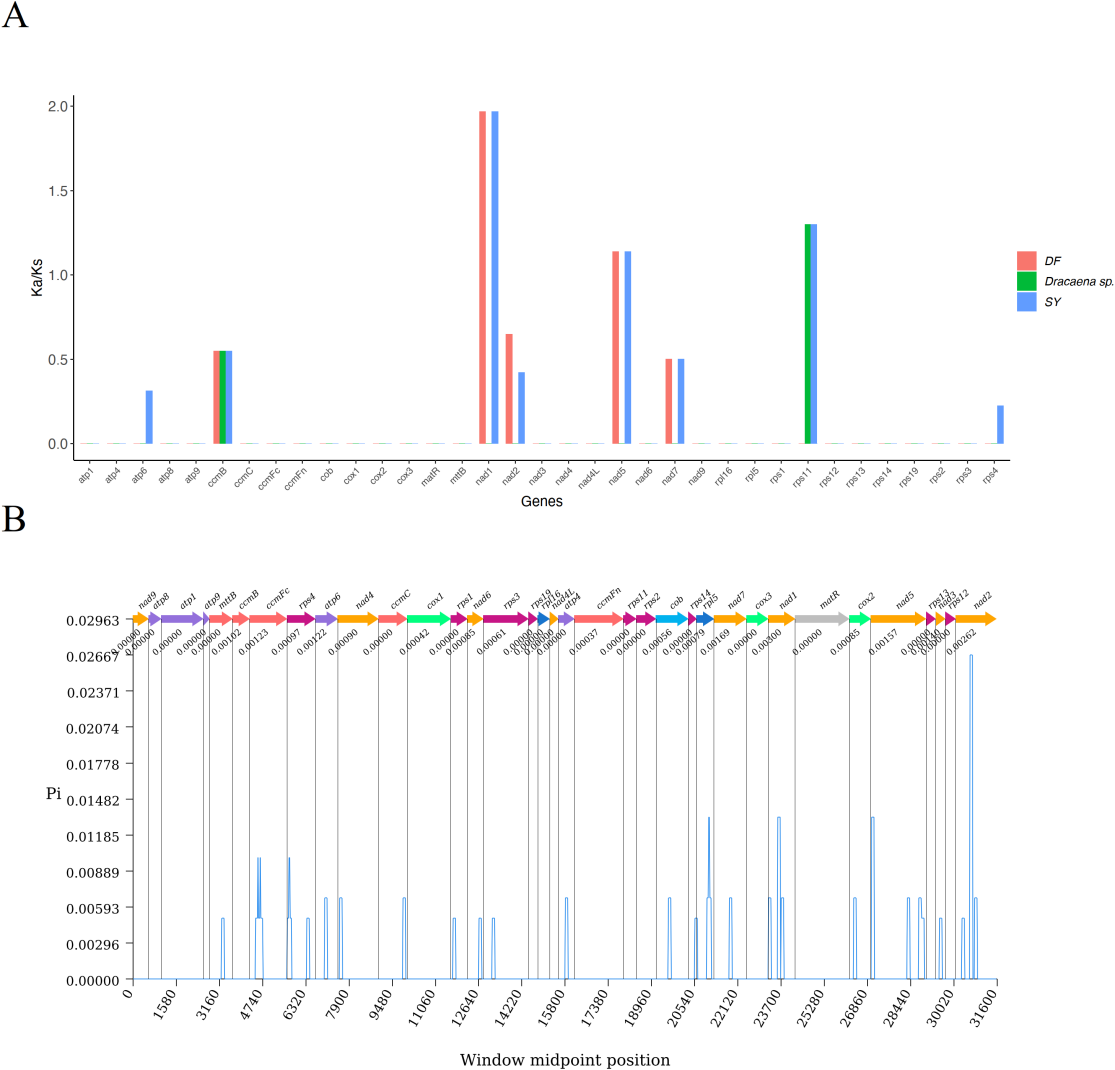
Variation in mitochondrial genes and the evolutionary characteristics of Dracaena. **(A)**. Ka/Ks ratio calculated for the CDSs. **(B)** nucleotide diversity of the CDSs.

To assess selective pressures on CDSs during evolutionary dynamics of *Dracaena*, we calculated the nonsynonymous (Ka) and synonymous (Ks) substitution ratios (Ka/Ks). A Ka/Ks ratio of 1 indicates neutral selection, where Ka equals Ks. A ratio greater than 1 (Ka/Ks > 1) suggests positive selection, while a ratio less than 1 (Ka/Ks < 1) indicates negative selection (Hurst, 2002). As depicted in **Figure 7A**, the gene-specific Ka/Ks values ranged from 0.000 to 1.967. The *nad1*, *nad5*, and *rps11* genes exhibited the highest Ka/Ks value >1 in SY, signifying positive selection during evolution. In contrast, the majority of genes had Ka/Ks values of 0. Genes such as *atp6, ccmB, nad2, nad7*, and *rps4* had values less than 1, indicating negative selection. Among these, the *ccmB* gene had the lowest average Ka/Ks value of 0.550, consistently below 1, which indicated strong purifying selection and high conservation throughout the evolutionary process in *Dracaena*.

Nucleotide diversity (pi) measures the variation in nucleotide sequences among different species. Regions with higher nucleotide diversity can serve as potential molecular markers for population genetics studies. **Figure 7B** illustrated the nucleotide diversity (pi) of the CDSs across *Dracaena*. The pi values ranged from 0.000 to 0.003, with the majority being below 0.001. Among these genes, *nad1* exhibited the highest variability with a pi of 0.003, followed by *nad2* at 0.00262 and *nad7* at 0.00169. In contrast, 18 genes had a pi value of 0, indicating they were the most conserved. Overall, the nucleotide diversity among the CDSs in *Dracaena* species was relatively low.

### 3.6 Phylogenetic analysis

**Figure 8:**
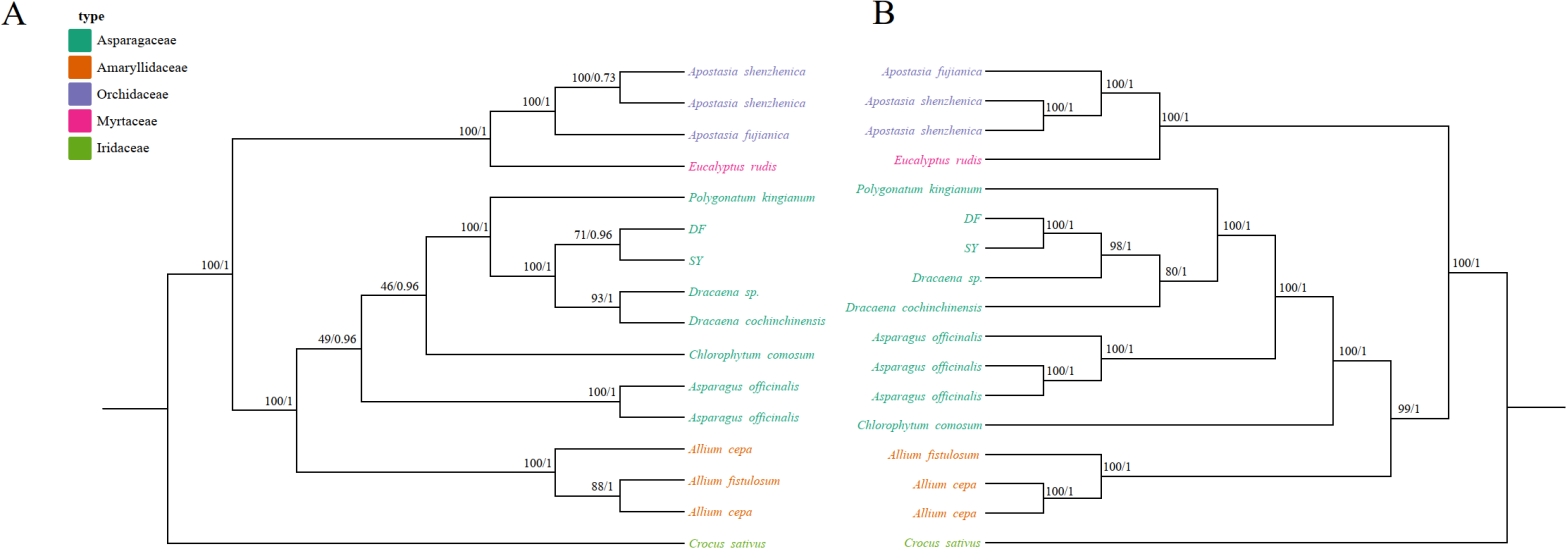
Molecular phylogenetic analysis using sequences from both mitochondrial and chloroplast ge-nomes. **(A)** A phylogenetic tree was generated using conserved protein sequences and analyzed with Maximum Likelihood (ML) and Bayesian Inference (BI) methods. The reliability of the tree was evaluated with bootstrap scores from 1000 replicates, with ML bootstrap support values and BI posterior probabilities indicated at the corresponding nodes. **(B)** The tree was constructed us-ing conserved protein sequences from the chloroplast genomes of the same species, applying the same methods as those used for the mitochondrial genome-based tree. genome-based tree.

We obtained the nucleotide sequences of mitochondrial CDSs from various plant species, including two *Dracaena*, three *Allium*, three *Apostasia*, two *Asparagus*, one *Chlorophytum*, one *Crocus*, one *Polygonatum*, and an outgroup *Eucalyptus rudis* (**Supplementary Table S9**). To analyze these sequences, we constructed phylogenetic trees using both Maximum Likelihood (ML) and Bayesian Inference (BI) methods. (**Figure 8**). In the phylogenetic tree, 9 out of 14 nodes showed bootstrap support values greater than 80% and posterior probabilities of 1. However, within the Asparagaceae clade, only 2 out of 7 nodes met these criteria (**Figure 8A**). Within this clade, *Asparagus* formed the basal group with a bootstrap support (BS) of 100 and a posterior probability (PP) of 1. Additionally, *Polygonatum kingianum* clustered closely with *Dracaena* species, also with high support (BS=100 and PP=1). DF and SY cluster into one clade (BS=100 and PP=1).

To compare the evolutionary processes of mitochondrial and chloroplast genes, we obtained chloroplast genome sequences of the same species from GenBank. We performed phylogenetic analysis on the sequences of CDSs using the same methods as those applied to the mitochondrial genome analysis (**Figure 8B**). We obtained a tree with a topological structure distinct from the one derived from mitochondrial CDSs. In this phylogenetic tree, 14 out of 15 nodes exhibited higher support (BS > 80 and PP = 1) compared to the mitochondrial dataset. Within the Asparagaceae species, *Chlorophytum comosum* formed the basal clade (BS = 100 and PP = 1), differing from **Figure 8A**. The *Dracaena* species also clustered into a single branch (BS = 100 and PP = 1). The trees constructed using mitochondrial and chloroplast sequences showed consistency.

## 4 Discussion

### 4.1 Variation in organelle genomes of *D. cambodiana* across contrasting habitats

In our study, we successfully assembled four organelle genomes from two *D. cambodiana* samples (collected from the coastal area and tropical forests) in Hainan Island. The “3 + 2” method are observed to form an integrated closed-loop structure of organelle genome (Liang et al., 2025b). Compared to the chloroplast genome, mitochondrial genomes are longer and have a more complex structure. However, the number of annotated genes in mitochondrial genome were less than in chloroplast genome. The identification of several intergenic hotspot regions (e.g., *trnC-GCA–petN*, *trnL-UAA–trnF-GAA*, *psaI–ycf4*, and *psbK–psbI*) between the DF and SY populations of *D. cambodiana* is consistent with previous studies in *Dracaena*, suggesting their potential as informative markers for species and population-level identification (Celiński et al., 2020). The mitochondrial genome sequences of the two *D. cambodiana* ranged in size from 521, 845 bp (DF) to 521,897 bp (SY), with the same GC content 49.66%. These values are consistent with those found in other Asparagaceae species, such as *Polygonatum kingianum* (555,557 bp). Exploring the mitochondrial genome could provide valuable insights into how to adapt to the environment and aid in refining taxonomic classifications in plant. By analyzing the structure and size of the mitochondrial genome across different *D. cambodiana*, it may be possible to infer the local adaptation mechanism and assess the rate of species divergence.

Analyses of nuclear, mitochondrial and chloroplast genomes have revealed that genes can move between different genomes within a cell (Korpelainen, 2004; Petit et al., 2005). This gene transfer, particularly between mitochondria and chloroplasts, is believed to play a role in long-term plant evolution (Adams et al., 2002; Møller et al., 2021).In most angiosperms, tRNA genes are often transferred from chloroplasts to the mitochondrial genome (Wang et al., 2007). In our study, we identified 25 and 26 fragments of chloroplast genomes within the mitochondrial genomes of DF and SY, respectively. Additionally, we found that tRNA genes: like *trnR-ACG* from chloroplast and *trnW-CCA* from mitochondrial and one CDS gene *rpl16* (partical) were only discovered in SY. Although the function of migrated DNA remains unclear, as do the motivations and mechanisms driving this phenomenon, further exploration of intergenomic transfer (IGT) could provide valuable insights into molecular evolution and establish a foundation for future genetic engineering efforts aimed at facilitating gene transfer between organelles. Therefore, the three genes will provide new insight into island conservation biology research. Further examination of organelle genomes of *D. cambodiana* will enhance our understanding of evolutionary processes. Previous studies have suggested that two significant horizontal gene transfer (HGT) events took place in the ancestors of land plants, likely driven by interspecies hybridization and frequent natural grafting (Stegemann et al., 2012; Ma et al., 2022). Although functional transfers from chloroplast to mitochondrial DNA are currently limited to tRNA genes, the addition of these chloroplast-derived sequences will undoubtedly contribute to the increasing complexity of the mitochondrial genome. Hence, the overall organellar genome structures of *D. cambodiana* from both regions are highly conserved, differences in hotspot regions and RNA editing sites likely reflect ecological adaptation.

### 4.2 Evolution of the organelle genome in *D. cambodiana*

Mitochondria are organelles in eukaryotic cells that have their own genetic system and are responsible for energy production through RNA expression and protein synthesis. In numerous plant species, mitogenomes are usually seen as a single circular molecule without isoforms (Li et al., 2021). However, with advancements in sequencing technology particularly third-generation sequencing and assembly software, numerous complex structures have been discovered in plant mitochondrial genomes, including combinations of master circular molecules, subgenomic circular molecules, linear molecules, and highly branched multigenomic structures (Zhou et al., 2023a, b). Our results suggest that the *D. cambodiana* mitochondrial genome is a circular molecule, which not a multipartite model of the mitochondrial genome. The structures of these two mitogenomes of *D. cambodiana* that in extreme environment is highly conservative, which indicates that environmental changes may have little effect on the structural changes of mitogenomes.

Numerous studies have shown that organelle genome is a useful and prospective tool for recovering the phylogeny of family or genus complex (Park et al., 2024). Moreover, the bootstrap support and posterior probability in chloroplast genome tree were higher than mitogenome, with almost universal topology. The phylogenetic clustering indicated that the dataset of organelle genome could be used to infer the complex phylogeny of Asparagaceae.

Moreover, several intergenic “hotspot” regions such as *trnC-GCA–petN*, *trnL-UAA–trnF-GAA*, *psaI–ycf4*, and *psbK–psbI* are known to be highly variable non-coding sequences, which are sensitive to evolutionary pressures and frequently involved in regulatory functions, potentially affecting gene expression and genome stability (Li et al., 2024). The biological significance of these “hotspot” regions likely relates to their roles in local adaptation processes; variations in these intergenic sequences may influence the efficiency of transcription, RNA processing, and gene expression regulation under environmental stresses (Ni et al., 2022). Specifically, differential evolution in these regions could lead to adaptive shifts in chloroplast function, such as optimizing photosynthetic efficiency and stress resilience under diverse ecological conditions like salt stress, drought, UV exposure, or temperature fluctuations (Fernández-Mazuecos and Vargas, 2010). Due to their high variability and sensitivity to environmental factors, these hotspot regions represent promising molecular markers for taxonomy, population genetics, phylogeography, and conservation biology. Further research incorporating more *Dracaena* population and experimental validation will be important to assess their effectiveness and functional relevance in ecological adaptation. This will deepen our understanding of their roles in chloroplast evolution and species diversification within island ecosystems.

RNA editing, primarily occurring in the coding transcripts of plant organelles, is believed to play a role in regulating gene expression (Gray and Covello, 1993). Numerous studies have shown that abnormal RNA editing leads to impaired organelle biogenesis, reduced embryo and endosperm development, stunted plant growth, and decreased adaptability to abiotic stresses (Jia et al., 2020; Lin et al., 2023; Liu et al., 2024; Saeedi et al., 2024). Plant organelle RNA editing has certain characteristics and machinery, which indicate RNA editing of mitogenome plays a vital role in the Conservation Biology of plants (Ramadan et al., 2024). During early plant terrestrialization, RNA editing mechanisms likely played a crucial role in reducing DNA damage caused by UV light, especially in the absence of protective systems. As this strong natural selection pressure diminished, more evolved species may have experienced “constructive neutral evolution,” leading to significant variations in RNA editing events (Small et al., 2020). In our study, there was little difference in the number of RNA editing sites and transfer type in DF and SY, the RNA editing even might not was affected the main reason for local adaptation of *D. cambodiana*. The molecular mechanism of how signals are relayed from organelles to the nucleus in response to stress remains one of the least understood signaling pathways in plants, even though it is of fundamental importance. Due to lack of *D. cambodiana* reference genome, we can not explain further.

In addition to structural and sequence-level comparisons, our results revealed important genomic features that offer insights into organelle genome evolution in *D. cambodiana*. The codon usage analysis showed a clear bias toward A/T-ending codons, particularly in leucine and isoleucine codons, which is consistent with patterns observed in many angiosperms. This may reflect both mutational bias in the organelle genomes and selection for translational efficiency. The highly similar codon usage profiles between the DF and SY populations suggest strong conservation of translational machinery in different habitats. Repetitive sequences, including SSRs and tandem repeats, were abundant in mitochondrial genomes. These elements are known to promote recombination and structural rearrangements, potentially contributing to organelle genome plasticity and evolution. Such features may also be hotspots for variation useful in population genetics. Moreover, the synteny analysis revealed several homologous segments between chloroplast and mitochondrial genomes (i.e., MTPTs), indicating potential past intergenomic transfer events. These syntenic regions may carry functional implications, such as shared tRNA genes or fragments involved in essential cellular functions. Together, these observations enhance our understanding of genome organization, stability, and evolution in *D. cambodiana* and reinforce the broader significance of comparative organelle genomics in evolutionary and ecological studies.

### 4.3 Stress-driven adaptive selection in *D. cambodiana* mitochondrial genes

The Ka/Ks analysis provides detailed insights into the evolution of plant mitochondrial genes (Wang et al., 2024). Most mitochondrial genes are generally highly conserved and have undergone neutral or negative selection. However, we detected three genes (*nad1*, *nad5* and *rps11*) with Ka/Ks values greater than 1.0, suggesting that these genes might have undergone positive selection during the evolution in *D. cambodiana* from the coastal area (SY). They are involved in the molecular functions of proton transmembrane transport and protein transmembrane transport driven by proton motive force. Gene *nad1* and *nad5* are the NADH dehydrogenase gene, which is a flavoprotein containing iron-sulfur centers and essential in the electron transport chain for ATP production; and *rps11* is the Ribosomal proteins (SSU) gene, which is involved in the protein synthesis process. Owing to the effects of frequent human activities and the harsh costal climate, these three genes might have developed novel functions for stress resistance in *D. cambodiana* plants under positive selected pressure. The SY population from the coastal area experiences long-term high salt, intense radiation, and drought stresses, which may have driven positive selection on the three genes. This positive selection potentially enhances metabolic efficiency and protein synthesis under extreme environmental stress. In contrast, the relatively stable environment in the DF region is correlated with higher genome stability, suggesting that genomic variability is closely linked to ecological stress intensity.

### 4.4 Conservation implications inferred from organelle genomic variation in *D. cambodiana*

In this study, we characterized and compared the organelle genomes of two *D. cambodiana* populations from ecologically distinct habitats on Hainan Island—one from a coastal area (SY) and the other from a mountainous tropical forest (DF). While the overall organelle genome structures were highly conserved, we identified several subtle but meaningful differences, including variations in RNA editing sites, intergenic hotspot regions, and positively selected genes (e.g., *nad1*, *nad5*, and *rps11*). These genes are functionally related to energy metabolism and protein synthesis and may contribute to the adaptation of the SY population to its harsher environment, which is characterized by high salinity, intense ultraviolet radiation, and prolonged drought conditions. In contrast, the DF population resides in a more stable, forested habitat with lower environmental stress and minimal human disturbance, resulting in fewer signs of selection and greater genomic stability. These genetic differences may reflect local adaptation to environmental pressures, and align with previous field observations that show poor regeneration and lower seedling survival in the SY population compared to DF. Thus, organelle genomic data can offer important insights into ecological resilience and vulnerability, supporting more targeted conservation approaches.

Although this study focused on only two populations, they were intentionally chosen to represent environmental extremes in *D. cambodiana*’s distribution. Our approach was to examine whether ecological differences are reflected in the organellar genomes. Nevertheless, we acknowledge the need to include more populations across Hainan in future studies to validate and refine these findings. Broader genomic sampling will be critical to support comprehensive conservation strategies.

From a conservation perspective, our findings have several implications. The SY population, under higher stress and showing signs of adaptive genomic variation, should be prioritized for *in-situ* conservation to preserve its unique ecological adaptations. Simultaneously, due to the environmental degradation and poor natural regeneration in both SY and DF populations, *ex-situ* conservation efforts, such as propagation and reintroduction, are also warranted. Previous demographic research has shown that *D. cambodiana* populations exhibit a Type I Deevey survival curve, with high mortality in early and late life stages and stability in the middle (Zheng et al., 2012). The current microhabitats, especially in SY, are unsuitable for seed germination due to increased exposure, drought, and human activities, which may accelerate endangerment. Genetic evidence from this study, particularly genes like *nad1*, *nad2*, and *nad7* with high nucleotide diversity, could serve as molecular markers for assessing the adaptive capacity of populations and guiding conservation actions. Ultimately, integrating genomic data with ecological monitoring will be crucial for developing dynamic, evidence-based conservation strategies for *D. cambodiana.*

## 5 Conclusion

The structural differences in organellar genomes between two *D. cambodiana.* from distinct habitats, highlighting potential ecological adaptation mechanisms. The positive selection observed in *nad1*, *nad5*, and *rps11* genes in the coastal SY population indicates adaptation to extreme ecological stress. The identification of these key genes and their ecological adaptive potential provides essential guidance for future conservation strategies and genetic diversity preservation measures. Furthermore, the hotspot regions identified can serve as valuable genetic markers for conservation monitoring of this species. While these findings suggest potential local adaptation at the organelle genome level, further ecological and physiological validation is required. Nonetheless, the identification of positively selected genes and variable intergenic regions offers a preliminary but valuable foundation for developing molecular markers and guiding conservation strategies. Future research should expand genomic sampling across additional populations and integrate environmental data to better elucidate the evolutionary dynamics and adaptive potential of *D. cambodiana*.

## Data Availability

The datasets presented in this study can be found in online repositories. The names of the repository/repositories and accession number(s) can be found in the article/**Supplementary Material**.
