# Supplementary material for: Comparative analysis of mitochondrial and chloroplast genomes of Dracaena cambodiana from contrasting island habitats
Source: Front Plant Sci. 2025 Jun 25;16:1620721. doi: 10.3389/fpls.2025.1620721 (PMC12237934; doi:10.3389/fpls.2025.1620721)
Supplement: Supplementary file 1 [file DataSheet1.docx]

Supplementary Material

# Supplementary Data

## Supplementary Figures

**C**

**Figure S1. The different growing condition of (A) DF and (B) SY and the distribution of DF and SY**

## Supplementary Tables

**TableS1 The protein-coding genes of the chloroplast genome of DF and SY**

| Category | Gene group | Gene name |
| --- | --- | --- |
| Photosynthesis | Subunits of photosystem I | *psaA,psaB,psaC,psaI,psaJ* |
|  | Subunits of photosystem II | *psbA,psbB,psbC,psbD,psbE,psbF,psbH,psbI,psbJ,psbK,psbL,psbM,psbN,psbT,psbZ* |
|  | Subunits of NADH dehydrogenase | *ndhA*,ndhB*(2),ndhC,ndhD,ndhE,ndhF,ndhG,ndhH,ndhI,ndhJ,ndhK* |
|  | Subunits of cytochrome b/f complex | *petA,petB*,petD*,petG,petL,petN* |
|  | Subunits of ATP synthase | *atpA,atpB,atpE,atpF*,atpH,atpI* |
|  | Large subunit of rubisco | *rbcL* |
|  | Subunits photochlorophyllide reductase | *-* |
| Self-replication | Proteins of large ribosomal subunit | *rpl14,rpl16*,rpl2*(2),rpl20,rpl22,rpl23(2),rpl32,rpl33,rpl36* |
|  | Proteins of small ribosomal subunit | *rps11,rps12**(2),rps14,rps15,rps16*,rps18,rps19(2),rps2,rps3,rps4,rps7(2),rps8* |
|  | Subunits of RNA polymerase | *rpoA,rpoB,rpoC1*,rpoC2* |
|  | Ribosomal RNAs | *rrn16(2),rrn23(2),rrn4.5(2),rrn5(2)* |
|  | Transfer RNAs | *trnA-UGC*(2),trnC-GCA,trnD-GUC,trnE-UUC,trnF-GAA,trnG-GCC,trnG-UCC*,trnH-GUG(2),trnI-CAU(2),trnI-GAU*(2),trnK-UUU*,trnL-CAA(2),trnL-UAA*,trnL-UAG,trnM-CAU,trnN-GUU(2),trnP-UGG,trnQ-UUG,trnR-ACG(2),trnR-UCU,trnS-GCU,trnS-GGA,trnS-UGA,trnT-GGU,trnT-UGU,trnV-GAC(2),trnV-UAC*,trnW-CCA,trnY-GUA,trnfM-CAU* |
| Other genes | Maturase | *#matK* |
|  | Protease | *clpP*** |
|  | Envelope membrane protein | *cemA* |
|  | Acetyl-CoA carboxylase | *accD* |
|  | c-type cytochrome synthesis gene | *ccsA* |
|  | Translation initiation factor | *#infA* |
|  | other | *-* |
| Genes of unknown function | Conserved hypothetical chloroplast ORF | *#ycf1,ycf1,ycf2(2),ycf3**,ycf4* |

Notes: Gene*:Gene with one introns; Gene**:Gene with two introns; #Gene:Pseudo gene;Gene(2):Number of copies of multi-copy genes;

**TableS2 The protein-coding genes of the mitochondrial genome of DF and SY**

| Group of genes | Gene name |
| --- | --- |
| ATP synthase | *atp1 atp4 atp6 atp8 atp9* |
| Cytohrome c biogenesis | *ccmB ccmC ccmFc* ccmFn* |
| Ubichinol cytochrome c reductase | *cob* |
| Cytochrome c oxidase | *cox1 cox2** cox3* |
| Maturases | *matR* |
| Transport membrance protein | *mttB* |
| NADH dehydrogenase | *nad1**** nad2**** nad3 nad4*** nad4L nad5**** nad6 nad7**** nad9* |
| Ribosomal proteins (LSU) | *rpl16 rpl5* |
| Ribosomal proteins (SSU) | *#rps7 rps1 rps10* rps11 rps12 rps13 rps14 rps19 rps2 rps3* rps4* |
| Succinate dehydrogenase |  |
| Ribosomal RNAs | *rrn18 rrn26 rrn5* |
| Transfer RNAs | *trnC-GCA(2) trnD-GTC trnE-TTC trnF-GAA trnG-GCC trnH-GTG trnK-TTT(3) trnM-CAT(5) trnN-GTT trnP-GGG trnP-TGG trnQ-TTG(2) trnS-GCT trnV-GAC trnW-CCA trnY-GTA* |
| other |  |

Notes:*:intron number;#Gene:Pseudo gene;Gene(2):Number of copies of multi-copy genes;

**TableS3A Prediction of RNA editing sites in DF**

| Type | RNA-editing | Number |
| --- | --- | --- |
| hydrophilic-hydrophilic | CAC (H) => TAC (Y) | 9 |
|  | CAT (H) => TAT (Y) | 20 |
|  | CGC (R) => TGC (C) | 12 |
|  | CGT (R) => TGT (C) | 34 |
|  | total | 75 |
| hydrophilic-hydrophobic | ACA (T) => ATA (I) | 5 |
|  | ACC (T) => ATC (I) | 6 |
|  | ACG (T) => ATG (M) | 7 |
|  | ACT (T) => ATT (I) | 4 |
|  | CGG (R) => TGG (W) | 39 |
|  | TCA (S) => TTA (L) | 75 |
|  | TCC (S) => TTC (F) | 41 |
|  | TCG (S) => TTG (L) | 50 |
|  | TCT (S) => TTT (F) | 55 |
|  | total | 282 |
| hydrophilic-stop | CAA (Q) => TAA (X) | 1 |
|  | CGA (R) => TGA (X) | 3 |
|  | total | 4 |
| hydrophobic-hydrophilic | CCA (P) => TCA (S) | 8 |
|  | CCC (P) => TCC (S) | 15 |
|  | CCG (P) => TCG (S) | 7 |
|  | CCT (P) => TCT (S) | 21 |
|  | total | 51 |
| hydrophobic-hydrophobic | CCA (P) => CTA (L) | 51 |
|  | CCC (P) => CTC (L) | 13 |
|  | CCC (P) => TTC (F) | 4 |
|  | CCG (P) => CTG (L) | 34 |
|  | CCT (P) => CTT (L) | 27 |
|  | CCT (P) => TTT (F) | 12 |
|  | CTC (L) => TTC (F) | 6 |
|  | CTT (L) => TTT (F) | 20 |
|  | GCC (A) => GTC (V) | 1 |
|  | GCG (A) => GTG (V) | 5 |
|  | GCT (A) => GTT (V) | 4 |
|  | total | 177 |
|  | All | 589 |

**TableS3B Prediction of RNA editing sites in SY**

| Type | RNA-editing | Number |
| --- | --- | --- |
| hydrophilic-hydrophilic | CAC (H) => TAC (Y) | 9 |
|  | CAT (H) => TAT (Y) | 20 |
|  | CGC (R) => TGC (C) | 12 |
|  | CGT (R) => TGT (C) | 34 |
|  | total | 75 |
| hydrophilic-hydrophobic | ACA (T) => ATA (I) | 5 |
|  | ACC (T) => ATC (I) | 5 |
|  | ACG (T) => ATG (M) | 6 |
|  | ACT (T) => ATT (I) | 4 |
|  | CGG (R) => TGG (W) | 39 |
|  | TCA (S) => TTA (L) | 75 |
|  | TCC (S) => TTC (F) | 42 |
|  | TCG (S) => TTG (L) | 49 |
|  | TCT (S) => TTT (F) | 54 |
|  | total | 279 |
| hydrophilic-stop | CAA (Q) => TAA (X) | 1 |
|  | CGA (R) => TGA (X) | 3 |
|  | total | 4 |
| hydrophobic-hydrophilic | CCA (P) => TCA (S) | 8 |
|  | CCC (P) => TCC (S) | 15 |
|  | CCG (P) => TCG (S) | 7 |
|  | CCT (P) => TCT (S) | 21 |
|  | total | 51 |
| hydrophobic-hydrophobic | CCA (P) => CTA (L) | 52 |
|  | CCC (P) => CTC (L) | 14 |
|  | CCC (P) => TTC (F) | 4 |
|  | CCG (P) => CTG (L) | 34 |
|  | CCT (P) => CTT (L) | 26 |
|  | CCT (P) => TTT (F) | 12 |
|  | CTC (L) => TTC (F) | 6 |
|  | CTT (L) => TTT (F) | 20 |
|  | GCC (A) => GTC (V) | 1 |
|  | GCG (A) => GTG (V) | 6 |
|  | GCT (A) => GTT (V) | 4 |
|  | total | 179 |
|  | All | 588 |

**TableS4 Relative synonymous codon usage values of DF and SY**

| AminoAcid | Symbol | Codon | No. | RSCU | Codon | No. | RSCU |
| --- | --- | --- | --- | --- | --- | --- | --- |
|  |  | DF | | | SY | | |
| * | Ter | UAA | 12 | 1.0588 | UAA | 12 | 1 |
| * | Ter | UAG | 6 | 0.5294 | UAG | 6 | 0.5 |
| * | Ter | UGA | 16 | 1.4118 | UGA | 18 | 1.5 |
| A | Ala | GCA | 157 | 0.9767 | GCA | 157 | 0.9767 |
| A | Ala | GCC | 153 | 0.9518 | GCC | 153 | 0.9518 |
| A | Ala | GCG | 86 | 0.535 | GCG | 86 | 0.535 |
| A | Ala | GCU | 247 | 1.5365 | GCU | 247 | 1.5365 |
| C | Cys | UGC | 59 | 0.7919 | UGC | 59 | 0.7867 |
| C | Cys | UGU | 90 | 1.2081 | UGU | 91 | 1.2133 |
| D | Asp | GAC | 110 | 0.6452 | GAC | 110 | 0.6433 |
| D | Asp | GAU | 231 | 1.3548 | GAU | 232 | 1.3567 |
| E | Glu | GAA | 294 | 1.3125 | GAA | 295 | 1.317 |
| E | Glu | GAG | 154 | 0.6875 | GAG | 153 | 0.683 |
| F | Phe | UUC | 291 | 0.8832 | UUC | 291 | 0.8832 |
| F | Phe | UUU | 368 | 1.1168 | UUU | 368 | 1.1168 |
| G | Gly | GGA | 268 | 1.4565 | GGA | 268 | 1.4565 |
| G | Gly | GGC | 101 | 0.5489 | GGC | 101 | 0.5489 |
| G | Gly | GGG | 122 | 0.663 | GGG | 122 | 0.663 |
| G | Gly | GGU | 245 | 1.3315 | GGU | 245 | 1.3315 |
| H | His | CAC | 63 | 0.4649 | CAC | 63 | 0.4649 |
| H | His | CAU | 208 | 1.5351 | CAU | 208 | 1.5351 |
| I | Ile | AUA | 206 | 0.7903 | AUA | 206 | 0.7893 |
| I | Ile | AUC | 226 | 0.867 | AUC | 226 | 0.8659 |
| I | Ile | AUU | 350 | 1.3427 | AUU | 351 | 1.3448 |
| K | Lys | AAA | 262 | 1.1542 | AAA | 262 | 1.1542 |
| K | Lys | AAG | 192 | 0.8458 | AAG | 192 | 0.8458 |
| L | Leu | CUA | 145 | 0.8254 | CUA | 144 | 0.8205 |
| L | Leu | CUC | 133 | 0.7571 | CUC | 132 | 0.7521 |
| L | Leu | CUG | 110 | 0.6262 | CUG | 111 | 0.6325 |
| L | Leu | CUU | 222 | 1.2638 | CUU | 222 | 1.265 |
| L | Leu | UUA | 240 | 1.3662 | UUA | 240 | 1.3675 |
| L | Leu | UUG | 204 | 1.1613 | UUG | 204 | 1.1624 |
| M | Met | AUG | 281 | 1 | AUG | 284 | 1 |
| N | Asn | AAC | 101 | 0.6474 | AAC | 101 | 0.6474 |
| N | Asn | AAU | 211 | 1.3526 | AAU | 211 | 1.3526 |
| P | Pro | CCA | 169 | 1.1229 | CCA | 170 | 1.1296 |
| P | Pro | CCC | 134 | 0.8904 | CCC | 134 | 0.8904 |
| P | Pro | CCG | 99 | 0.6578 | CCG | 99 | 0.6578 |
| P | Pro | CCU | 200 | 1.3289 | CCU | 199 | 1.3223 |
| Q | Gln | CAA | 217 | 1.5445 | CAA | 217 | 1.5445 |
| Q | Gln | CAG | 64 | 0.4555 | CAG | 64 | 0.4555 |
| R | Arg | AGA | 168 | 1.3263 | AGA | 168 | 1.3298 |
| R | Arg | AGG | 85 | 0.6711 | AGG | 85 | 0.6728 |
| R | Arg | CGA | 170 | 1.3421 | CGA | 168 | 1.3298 |
| R | Arg | CGC | 74 | 0.5842 | CGC | 74 | 0.5858 |
| R | Arg | CGG | 104 | 0.8211 | CGG | 104 | 0.8232 |
| R | Arg | CGU | 159 | 1.2553 | CGU | 159 | 1.2586 |
| S | Ser | AGC | 94 | 0.5618 | AGC | 94 | 0.5623 |
| S | Ser | AGU | 165 | 0.9861 | AGU | 164 | 0.9811 |
| S | Ser | UCA | 191 | 1.1414 | UCA | 191 | 1.1426 |
| S | Ser | UCC | 180 | 1.0757 | UCC | 180 | 1.0768 |
| S | Ser | UCG | 148 | 0.8845 | UCG | 148 | 0.8853 |
| S | Ser | UCU | 226 | 1.3506 | UCU | 226 | 1.3519 |
| T | Thr | ACA | 139 | 1.0335 | ACA | 138 | 1.0318 |
| T | Thr | ACC | 141 | 1.0483 | ACC | 141 | 1.0542 |
| T | Thr | ACG | 85 | 0.632 | ACG | 82 | 0.6131 |
| T | Thr | ACU | 173 | 1.2862 | ACU | 174 | 1.3009 |
| V | Val | GUA | 185 | 1.1473 | GUA | 184 | 1.1411 |
| V | Val | GUC | 133 | 0.8248 | GUC | 134 | 0.831 |
| V | Val | GUG | 148 | 0.9178 | GUG | 148 | 0.9178 |
| V | Val | GUU | 179 | 1.1101 | GUU | 179 | 1.1101 |
| W | Trp | UGG | 144 | 1 | UGG | 144 | 1 |
| Y | Tyr | UAC | 71 | 0.4686 | UAC | 70 | 0.4636 |
| Y | Tyr | UAU | 232 | 1.5314 | UAU | 232 | 1.5364 |

**TableS5 SSR statistics of DF and SY**

| SSR nr. | SSR type | SSR | size | start | end | SSR nr. | SSR type | SSR | size | start | end |
| --- | --- | --- | --- | --- | --- | --- | --- | --- | --- | --- | --- |
|  | DF | | | | | SY | | | | | |
| 1 | p1 | (T)13 | 13 | 1433 | 1445 | 1 | p1 | (T)13 | 13 | 1433 | 1445 |
| 2 | p1 | (A)11 | 11 | 2514 | 2524 | 2 | p1 | (A)11 | 11 | 2510 | 2520 |
| 3 | p1 | (T)10 | 10 | 3229 | 3238 | 3 | p1 | (T)10 | 10 | 3225 | 3234 |
| 4 | p4 | (GGAA)4 | 16 | 8383 | 8398 | 4 | p4 | (GGAA)4 | 16 | 8379 | 8394 |
| 5 | p4 | (GAAT)3 | 12 | 11008 | 11019 | 5 | p4 | (GAAT)3 | 12 | 11004 | 11015 |
| 6 | p3 | (CGA)4 | 12 | 11625 | 11636 | 6 | p3 | (CGA)4 | 12 | 11621 | 11632 |
| 7 | p4 | (TCAA)3 | 12 | 12250 | 12261 | 7 | p4 | (TCAA)3 | 12 | 12246 | 12257 |
| 8 | p1 | (G)11 | 11 | 15821 | 15831 | 8 | p1 | (G)11 | 11 | 15817 | 15827 |
| 9 | p4 | (ACTG)3 | 12 | 16226 | 16237 | 9 | p4 | (ACTG)3 | 12 | 16222 | 16233 |
| 10 | p1 | (A)10 | 10 | 16370 | 16379 | 10 | p1 | (A)10 | 10 | 16366 | 16375 |
| 11 | p1 | (T)10 | 10 | 24672 | 24681 | 11 | p1 | (T)10 | 10 | 24668 | 24677 |
| 12 | p2 | (AG)5 | 10 | 26622 | 26631 | 12 | p2 | (AG)5 | 10 | 26618 | 26627 |
| 13 | p3 | (TCT)4 | 12 | 30401 | 30412 | 13 | p3 | (TCT)4 | 12 | 30397 | 30408 |
| 14 | p2 | (AT)5 | 10 | 36562 | 36571 | 14 | p2 | (CT)5 | 10 | 37023 | 37032 |
| 15 | p2 | (CT)5 | 10 | 37029 | 37038 | 15 | p4 | (TCGG)3 | 12 | 38145 | 38156 |
| 16 | p4 | (TCGG)3 | 12 | 38151 | 38162 | 16 | p4 | (GCTA)3 | 12 | 39389 | 39400 |
| 17 | p4 | (GCTA)3 | 12 | 39403 | 39414 | 17 | p1 | (A)10 | 10 | 41035 | 41044 |
| 18 | p1 | (A)11 | 11 | 41049 | 41059 | 18 | p4 | (AAAG)3 | 12 | 48096 | 48107 |
| 19 | p4 | (AAAG)3 | 12 | 48111 | 48122 | 19 | p4 | (AAGA)3 | 12 | 52729 | 52740 |
| 20 | p4 | (AAGA)3 | 12 | 52744 | 52755 | 20 | p1 | (T)11 | 11 | 52829 | 52839 |
| 21 | p1 | (T)12 | 12 | 52844 | 52855 | 21 | p4 | (AGGA)3 | 12 | 53358 | 53369 |
| 22 | p4 | (AGGA)3 | 12 | 53374 | 53385 | 22 | p4 | (CTTT)3 | 12 | 53923 | 53934 |
| 23 | p4 | (CTTT)3 | 12 | 53939 | 53950 | 23 | p2 | (CT)5 | 10 | 56984 | 56993 |
| 24 | p2 | (CT)5 | 10 | 57000 | 57009 | 24 | p3 | (CTT)4 | 12 | 66478 | 66489 |
| 25 | p3 | (CTT)4 | 12 | 66494 | 66505 | 25 | p3 | (CTT)4 | 12 | 66640 | 66651 |
| 26 | p3 | (CTT)4 | 12 | 66656 | 66667 | 26 | p2 | (GA)7 | 14 | 81314 | 81327 |
| 27 | p2 | (GA)7 | 14 | 81331 | 81344 | 27 | p2 | (AG)5 | 10 | 93320 | 93329 |
| 28 | p2 | (AG)5 | 10 | 93337 | 93346 | 28 | p1 | (T)10 | 10 | 94301 | 94310 |
| 29 | p1 | (T)10 | 10 | 94318 | 94327 | 29 | p5 | (GGAAC)3 | 15 | 96493 | 96507 |
| 30 | p5 | (GGAAC)3 | 15 | 96510 | 96524 | 30 | p4 | (AAGA)3 | 12 | 103533 | 103544 |
| 31 | p4 | (AAGA)3 | 12 | 103552 | 103563 | 31 | p1 | (A)10 | 10 | 110256 | 110265 |
| 32 | p1 | (A)10 | 10 | 110273 | 110282 | 32 | p1 | (T)10 | 10 | 110570 | 110579 |
| 33 | p1 | (T)10 | 10 | 110587 | 110596 | 33 | p2 | (AG)5 | 10 | 112747 | 112756 |
| 34 | p2 | (AG)5 | 10 | 112764 | 112773 | 34 | p2 | (TC)5 | 10 | 115904 | 115913 |
| 35 | p2 | (TC)5 | 10 | 115921 | 115930 | 35 | p1 | (T)10 | 10 | 126580 | 126589 |
| 36 | p1 | (T)10 | 10 | 126597 | 126606 | 36 | p2 | (AG)5 | 10 | 137294 | 137303 |
| 37 | p2 | (AG)5 | 10 | 137311 | 137320 | 37 | p1 | (A)10 | 10 | 144400 | 144409 |
| 38 | p1 | (A)10 | 10 | 143715 | 143724 | 38 | p2 | (AG)5 | 10 | 144838 | 144847 |
| 39 | p1 | (A)10 | 10 | 144418 | 144427 | 39 | p1 | (A)10 | 10 | 149572 | 149581 |
| 40 | p2 | (AG)5 | 10 | 144856 | 144865 | 40 | p3 | (TCT)4 | 12 | 149742 | 149753 |
| 41 | p1 | (A)10 | 10 | 149590 | 149599 | 41 | p1 | (T)10 | 10 | 152709 | 152718 |
| 42 | p3 | (TCT)4 | 12 | 149760 | 149771 | 42 | p4 | (CTTT)3 | 12 | 159071 | 159082 |
| 43 | p1 | (T)10 | 10 | 152727 | 152736 | 43 | p1 | (T)10 | 10 | 159329 | 159338 |
| 44 | p4 | (CTTT)3 | 12 | 159089 | 159100 | 44 | p1 | (A)10 | 10 | 159378 | 159387 |
| 45 | p1 | (T)10 | 10 | 159347 | 159356 | 45 | p2 | (AC)5 | 10 | 162972 | 162981 |
| 46 | p1 | (A)10 | 10 | 159396 | 159405 | 46 | p4 | (GATC)3 | 12 | 166615 | 166626 |
| 47 | p2 | (AC)5 | 10 | 162990 | 162999 | 47 | p3 | (AAC)4 | 12 | 173812 | 173823 |
| 48 | p4 | (GATC)3 | 12 | 166633 | 166644 | 48 | p3 | (GGT)4 | 12 | 175075 | 175086 |
| 49 | p3 | (AAC)4 | 12 | 173830 | 173841 | 49 | p2 | (TC)5 | 10 | 175496 | 175505 |
| 50 | p3 | (GGT)4 | 12 | 175093 | 175104 | 50 | p1 | (A)10 | 10 | 177380 | 177389 |
| 51 | p2 | (TC)5 | 10 | 175514 | 175523 | 51 | p3 | (CAA)4 | 12 | 178120 | 178131 |
| 52 | p1 | (A)10 | 10 | 177398 | 177407 | 52 | p4 | (CATT)3 | 12 | 183582 | 183593 |
| 53 | p3 | (CAA)4 | 12 | 178138 | 178149 | 53 | p2 | (TC)5 | 10 | 183744 | 183753 |
| 54 | p4 | (CATT)3 | 12 | 183600 | 183611 | 54 | p4 | (TGAC)3 | 12 | 191967 | 191978 |
| 55 | p2 | (TC)5 | 10 | 183762 | 183771 | 55 | p1 | (A)10 | 10 | 195145 | 195154 |
| 56 | p4 | (TGAC)3 | 12 | 191985 | 191996 | 56 | p3 | (CAG)4 | 12 | 201233 | 201244 |
| 57 | p1 | (A)10 | 10 | 195163 | 195172 | 57 | p2 | (AG)5 | 10 | 206491 | 206500 |
| 58 | p3 | (CAG)4 | 12 | 201251 | 201262 | 58 | p1 | (T)10 | 10 | 213147 | 213156 |
| 59 | p2 | (AG)5 | 10 | 206509 | 206518 | 59 | p2 | (AG)6 | 12 | 218856 | 218867 |
| 60 | p1 | (T)10 | 10 | 213165 | 213174 | 60 | p5 | (CGGTA)3 | 15 | 219594 | 219608 |
| 61 | p2 | (AG)6 | 12 | 218874 | 218885 | 61 | p1 | (A)10 | 10 | 226017 | 226026 |
| 62 | p5 | (CGGTA)3 | 15 | 219612 | 219626 | 62 | p4 | (TTCC)3 | 12 | 227546 | 227557 |
| 63 | p1 | (A)10 | 10 | 226035 | 226044 | 63 | p1 | (T)10 | 10 | 229946 | 229955 |
| 64 | p4 | (TTCC)3 | 12 | 227564 | 227575 | 64 | p4 | (TGGT)3 | 12 | 232427 | 232438 |
| 65 | p1 | (T)10 | 10 | 229964 | 229973 | 65 | p3 | (CTC)4 | 12 | 236379 | 236390 |
| 66 | p3 | (CTC)4 | 12 | 236397 | 236408 | 66 | p3 | (CAA)5 | 15 | 238199 | 238213 |
| 67 | p3 | (CAA)5 | 15 | 238217 | 238231 | 67 | p6 | (TTTCAT)3 | 18 | 240894 | 240911 |
| 68 | p6 | (TTTCAT)3 | 18 | 240912 | 240929 | 68 | p4 | (AATG)3 | 12 | 242338 | 242349 |
| 69 | p4 | (AATG)3 | 12 | 242356 | 242367 | 69 | p4 | (TAAA)3 | 12 | 250556 | 250567 |
| 70 | p1 | (T)10 | 10 | 248756 | 248765 | 70 | p2 | (TC)5 | 10 | 254977 | 254986 |
| 71 | p4 | (TAAA)3 | 12 | 250571 | 250582 | 71 | p5 | (GGAGA)3 | 15 | 256074 | 256088 |
| 72 | p1 | (T)10 | 10 | 252405 | 252414 | 72 | p2 | (AG)5 | 10 | 260038 | 260047 |
| 73 | p2 | (AG)5 | 10 | 253245 | 253254 | 73 | p4 | (CTTT)3 | 12 | 265705 | 265716 |
| 74 | p1 | (T)10 | 10 | 261591 | 261600 | 74 | p4 | (TTTC)3 | 12 | 271845 | 271856 |
| 75 | p3 | (TGT)4 | 12 | 263562 | 263573 | 75 | p1 | (T)10 | 10 | 272735 | 272744 |
| 76 | p2 | (CT)6 | 12 | 265779 | 265790 | 76 | p5 | (AATCG)3 | 15 | 274510 | 274524 |
| 77 | p4 | (AGCC)3 | 12 | 267289 | 267300 | 77 | p1 | (T)10 | 10 | 285557 | 285566 |
| 78 | p4 | (TTCA)3 | 12 | 267417 | 267428 | 78 | p1 | (T)10 | 10 | 286417 | 286426 |
| 79 | p4 | (TGAC)3 | 12 | 274072 | 274083 | 79 | p2 | (CT)5 | 10 | 288734 | 288743 |
| 80 | p2 | (AT)7 | 14 | 277088 | 277101 | 80 | p1 | (T)10 | 10 | 293326 | 293335 |
| 81 | p4 | (CTTT)3 | 12 | 278105 | 278116 | 81 | p2 | (TC)5 | 10 | 301260 | 301269 |
| 82 | p3 | (GAA)4 | 12 | 278489 | 278500 | 82 | p4 | (GAAA)3 | 12 | 309119 | 309130 |
| 83 | p4 | (TGCC)3 | 12 | 279618 | 279629 | 83 | p5 | (TTTCG)3 | 15 | 315327 | 315341 |
| 84 | p3 | (CGA)4 | 12 | 283367 | 283378 | 84 | p2 | (CT)5 | 10 | 325938 | 325947 |
| 85 | p1 | (A)10 | 10 | 293966 | 293975 | 85 | p3 | (AGA)7 | 21 | 328172 | 328192 |
| 86 | p1 | (T)10 | 10 | 296055 | 296064 | 86 | p3 | (TGC)4 | 12 | 335493 | 335504 |
| 87 | p4 | (CTTC)3 | 12 | 302061 | 302072 | 87 | p5 | (GATAC)3 | 15 | 337134 | 337148 |
| 88 | p4 | (ATCA)3 | 12 | 304603 | 304614 | 88 | p1 | (T)10 | 10 | 339597 | 339606 |
| 89 | p4 | (ATGA)3 | 12 | 310157 | 310168 | 89 | p2 | (AG)5 | 10 | 340437 | 340446 |
| 90 | p2 | (AG)5 | 10 | 310877 | 310886 | 90 | p4 | (GTCA)3 | 12 | 351542 | 351553 |
| 91 | p2 | (CT)5 | 10 | 318614 | 318623 | 91 | p2 | (GA)5 | 10 | 351969 | 351978 |
| 92 | p2 | (AT)10 | 20 | 318817 | 318836 | 92 | p1 | (T)10 | 10 | 353904 | 353913 |
| 93 | p2 | (TC)5 | 10 | 324557 | 324566 | 93 | p3 | (AGA)4 | 12 | 361728 | 361739 |
| 94 | p2 | (CT)5 | 10 | 329947 | 329956 | 94 | p1 | (T)10 | 10 | 364130 | 364139 |
| 95 | p1 | (T)10 | 10 | 334539 | 334548 | 95 | p1 | (T)10 | 10 | 367044 | 367053 |
| 96 | p2 | (TC)5 | 10 | 342473 | 342482 | 96 | p2 | (GA)5 | 10 | 371502 | 371511 |
| 97 | p4 | (GAAA)3 | 12 | 350332 | 350343 | 97 | p2 | (AG)5 | 10 | 377433 | 377442 |
| 98 | p2 | (CT)5 | 10 | 367109 | 367118 | 98 | p2 | (TC)5 | 10 | 385169 | 385178 |
| 99 | p3 | (AGA)7 | 21 | 369344 | 369364 | 99 | p4 | (TCAT)3 | 12 | 385888 | 385899 |
| 100 | p3 | (TGC)4 | 12 | 376665 | 376676 | 100 | p4 | (TGAT)3 | 12 | 391442 | 391453 |
| 101 | p5 | (GATAC)3 | 15 | 378306 | 378320 | 101 | p4 | (GGAA)3 | 12 | 393983 | 393994 |
| 102 | p2 | (TC)5 | 10 | 383356 | 383365 | 102 | p1 | (A)10 | 10 | 399992 | 400001 |
| 103 | p5 | (GGAGA)3 | 15 | 384453 | 384467 | 103 | p3 | (TCG)4 | 12 | 412678 | 412689 |
| 104 | p2 | (AG)5 | 10 | 388417 | 388426 | 104 | p4 | (GGCA)3 | 12 | 416427 | 416438 |
| 105 | p4 | (CTTT)3 | 12 | 394084 | 394095 | 105 | p3 | (TTC)4 | 12 | 417556 | 417567 |
| 106 | p4 | (TTTC)3 | 12 | 400224 | 400235 | 106 | p4 | (AAAG)3 | 12 | 417940 | 417951 |
| 107 | p1 | (T)10 | 10 | 401114 | 401123 | 107 | p2 | (AT)6 | 12 | 418955 | 418966 |
| 108 | p1 | (T)10 | 10 | 413893 | 413902 | 108 | p4 | (GTCA)3 | 12 | 421971 | 421982 |
| 109 | p1 | (T)10 | 10 | 414753 | 414762 | 109 | p4 | (ATGA)3 | 12 | 428623 | 428634 |
| 110 | p1 | (A)10 | 10 | 416138 | 416147 | 110 | p4 | (GGCT)3 | 12 | 428752 | 428763 |
| 111 | p1 | (A)10 | 10 | 419052 | 419061 | 111 | p2 | (AG)6 | 12 | 430262 | 430273 |
| 112 | p3 | (TCT)4 | 12 | 421452 | 421463 | 112 | p3 | (CAA)4 | 12 | 432477 | 432488 |
| 113 | p1 | (A)10 | 10 | 429278 | 429287 | 113 | p1 | (A)10 | 10 | 434452 | 434461 |
| 114 | p2 | (TC)5 | 10 | 431213 | 431222 | 114 | p4 | (TAAA)3 | 12 | 450022 | 450033 |
| 115 | p4 | (TGAC)3 | 12 | 431638 | 431649 | 115 | p1 | (A)10 | 10 | 450031 | 450040 |
| 116 | p4 | (TAAA)3 | 12 | 449969 | 449980 | 116 | p5 | (GCGTC)3 | 15 | 453285 | 453299 |
| 117 | p1 | (A)10 | 10 | 449978 | 449987 | 117 | p4 | (GGAA)3 | 12 | 456412 | 456423 |
| 118 | p5 | (GCGTC)3 | 15 | 453232 | 453246 | 118 | p1 | (T)10 | 10 | 459790 | 459799 |
| 119 | p4 | (GGAA)3 | 12 | 456359 | 456370 | 119 | p2 | (AG)6 | 12 | 460202 | 460213 |
| 120 | p1 | (T)10 | 10 | 459737 | 459746 | 120 | p1 | (A)11 | 11 | 462676 | 462686 |
| 121 | p2 | (AG)6 | 12 | 460149 | 460160 | 121 | p1 | (G)10 | 10 | 470941 | 470950 |
| 122 | p1 | (G)10 | 10 | 470888 | 470897 | 122 | p4 | (TTTC)3 | 12 | 471223 | 471234 |
| 123 | p4 | (TTTC)3 | 12 | 471170 | 471181 | 123 | p4 | (AGAA)3 | 12 | 471736 | 471747 |
| 124 | p4 | (AGAA)3 | 12 | 471683 | 471694 | 124 | p2 | (GA)5 | 10 | 476650 | 476659 |
| 125 | p2 | (GA)5 | 10 | 476597 | 476606 | 125 | p5 | (AACAA)3 | 15 | 478872 | 478886 |
| 126 | p5 | (AACAA)3 | 15 | 478819 | 478833 | 126 | p2 | (AT)5 | 10 | 480169 | 480178 |
| 127 | p1 | (T)11 | 11 | 480010 | 480020 | 127 | p3 | (AAG)4 | 12 | 481742 | 481753 |
| 128 | p2 | (AT)5 | 10 | 480116 | 480125 | 128 | p2 | (AG)6 | 12 | 485328 | 485339 |
| 129 | p3 | (AAG)4 | 12 | 481689 | 481700 | 129 | p4 | (CTCC)3 | 12 | 492177 | 492188 |
| 130 | p2 | (AG)6 | 12 | 485275 | 485286 | 130 | p4 | (GCTC)3 | 12 | 499229 | 499240 |
| 131 | p4 | (CTCC)3 | 12 | 492124 | 492135 | 131 | p2 | (TC)5 | 10 | 506791 | 506800 |
| 132 | p4 | (GCTC)3 | 12 | 499176 | 499187 | 132 | p4 | (ATCC)3 | 12 | 510681 | 510692 |
| 133 | p2 | (TC)5 | 10 | 506738 | 506747 | 133 | p4 | (TTCC)3 | 12 | 513503 | 513514 |
| 134 | p4 | (ATCC)3 | 12 | 510628 | 510639 | 134 | p1 | (T)12 | 12 | 514723 | 514734 |
| 135 | p1 | (A)10 | 10 | 511683 | 511692 | 135 | p6 | (CGTCTA)3 | 18 | 519585 | 519602 |
| 136 | p4 | (TTCC)3 | 12 | 513451 | 513462 | 136 | p4 | (ATAG)3 | 12 | 520098 | 520109 |
| 137 | p1 | (T)12 | 12 | 514671 | 514682 |  |  |  |  |  |  |
| 138 | p6 | (CGTCTA)3 | 18 | 519533 | 519550 |  |  |  |  |  |  |
| 139 | p4 | (ATAG)3 | 12 | 520046 | 520057 |  |  |  |  |  |  |

**TableS6 Tandem repeats statistics of DF and SY**

| NO | Size | Copy | Repeat sequence | Percent Matches | Start | End | NO | Size | Copy | Repeat sequence | Percent Matches | Start | End |
| --- | --- | --- | --- | --- | --- | --- | --- | --- | --- | --- | --- | --- | --- |
|  | DF | | | | | | SY | | | | | | |
| 1 | 21 | 2.2 | TCCTTTGCTGATGCAGCGGA | 87 | 14390 | 14433 | 1 | 21 | 2.2 | TCCTTTGCTGATGCAGCGGA | 87 | 14386 | 14429 |
| 2 | 15 | 2.1 | CGGGATGCACTGACGT | 88 | 194773 | 194805 | 2 | 10 | 2.5 | ATATAAATAA | 100 | 108431 | 108455 |
| 3 | 17 | 2 | TTACGTCGAAGTACCAGC | 88 | 216262 | 216296 | 3 | 15 | 2.1 | CGGGATGCACTGACGT | 88 | 194755 | 194787 |
| 4 | 24 | 2 | ACTCATAGCACAGACGATCCGTCA | 87 | 229564 | 229611 | 4 | 17 | 2 | TTACGTCGAAGTACCAGC | 88 | 216244 | 216278 |
| 5 | 34 | 2.5 | GTCCCGCACTTCCCTATCAACAGGAGAGGAAACTT | 77 | 327408 | 327492 | 5 | 24 | 2 | ACTCATAGCACAGACGATCCGTCA | 87 | 229546 | 229593 |
| 6 | 17 | 2.4 | AGAAAGAGGTGATCGCCC | 80 | 371827 | 371869 | 6 | 28 | 2 | TCCAGACCATACAAATGCCCGTCATCGC | 100 | 252730 | 252786 |
| 7 | 28 | 2 | TCCAGACCATACAAATGCCCGTCATCGC | 100 | 381109 | 381165 | 7 | 33 | 2.4 | AGGGATTCCATCGGGCTGTAGCTATGCAGCTAC | 100 | 278909 | 278987 |
| 8 | 24 | 3.2 | CGGGGGCCCGGGCGGTGGAAGGTT | 96 | 435075 | 435151 | 8 | 17 | 2.4 | AGAAAGAGGTGATCGCCC | 80 | 330655 | 330697 |
| 9 | 17 | 2.2 | AAGTGATAGAATAGAGA | 95 | 440083 | 440119 | 9 | 24 | 3.2 | CCCCGAACCTTCCACCGCCCGGGC | 96 | 348040 | 348116 |
|  |  |  |  |  |  |  | 10 | 34 | 2.5 | TAGGGAAGTGCGGGACAAGATTCCTCTCCTGTTGA | 77 | 368576 | 368660 |
|  |  |  |  |  |  |  | 11 | 17 | 2.2 | AAGTGATAGAATAGAGA | 95 | 440136 | 440172 |

**TableS7 Dispersed repeats statistics of DF and SY**

| type | alignment length | similarity | start1 | end1 | start2 | end2 | evalue | type | alignment length | similarity | start1 | end1 | start2 | end2 | evalue |
| --- | --- | --- | --- | --- | --- | --- | --- | --- | --- | --- | --- | --- | --- | --- | --- |
| DF | | | | | | | | SY | | | | | | | |
| P | 5613 | 100 | 254809 | 260421 | 131350 | 136962 | 0 | P | 5617 | 100 | 342001 | 347617 | 131329 | 136945 | 0 |
| F | 4077 | 100 | 435574 | 439650 | 131346 | 135422 | 0 | F | 4073 | 100 | 435631 | 439703 | 131333 | 135405 | 0 |
| P | 4073 | 100 | 435578 | 439650 | 256349 | 260421 | 0 | P | 4073 | 100 | 435631 | 439703 | 343541 | 347613 | 0 |
| F | 3635 | 100 | 154154 | 157788 | 121793 | 125427 | 0 | F | 3635 | 100 | 154136 | 157770 | 121776 | 125410 | 0 |
| P | 2358 | 100 | 495940 | 498297 | 49116 | 51473 | 0 | P | 2358 | 100 | 495993 | 498350 | 49101 | 51458 | 0 |
| P | 2361 | 99.958 | 49116 | 51476 | 495937 | 498297 | 0 | P | 2361 | 99.958 | 49101 | 51461 | 495990 | 498350 | 0 |
| F | 2108 | 100 | 390651 | 392758 | 350657 | 352764 | 0 | F | 2109 | 100 | 309443 | 311551 | 262271 | 264379 | 0 |
| F | 468 | 92.735 | 415622 | 416088 | 328506 | 328965 | 0 | P | 468 | 92.735 | 367103 | 367562 | 287286 | 287752 | 0 |
| F | 576 | 84.722 | 273072 | 273620 | 95399 | 95967 | 1.28E-153 | P | 576 | 84.722 | 422434 | 422982 | 95382 | 95950 | 1.28E-153 |
| P | 277 | 98.917 | 520373 | 520649 | 421850 | 422123 | 1.70E-137 | P | 345 | 91.594 | 274097 | 274430 | 37100 | 37443 | 1.03E-129 |
| P | 345 | 91.594 | 402476 | 402809 | 37106 | 37449 | 1.03E-129 | F | 206 | 99.029 | 463373 | 463578 | 172200 | 172405 | 8.29E-101 |
| F | 206 | 99.029 | 463320 | 463525 | 172218 | 172423 | 8.29E-101 | F | 198 | 99.495 | 240642 | 240839 | 191521 | 191718 | 4.99E-98 |
| F | 198 | 99.495 | 240660 | 240857 | 191539 | 191736 | 4.99E-98 | F | 274 | 90.146 | 520425 | 520698 | 361068 | 361338 | 8.35E-96 |
| P | 178 | 98.876 | 419322 | 419499 | 336633 | 336810 | 3.04E-85 | F | 178 | 98.876 | 363692 | 363869 | 295420 | 295597 | 3.05E-85 |
| P | 178 | 98.876 | 336636 | 336813 | 419319 | 419496 | 3.04E-85 | P | 169 | 95.266 | 455766 | 455934 | 208771 | 208939 | 3.11E-70 |
| P | 169 | 95.266 | 455713 | 455881 | 208789 | 208957 | 3.11E-70 | P | 157 | 97.452 | 208783 | 208939 | 455766 | 455922 | 3.11E-70 |
| P | 157 | 97.452 | 208801 | 208957 | 455713 | 455869 | 3.11E-70 | F | 163 | 93.252 | 446066 | 446227 | 222671 | 222833 | 2.44E-61 |
| F | 163 | 93.252 | 446013 | 446174 | 222689 | 222851 | 2.44E-61 | F | 462 | 76.407 | 316167 | 316622 | 296053 | 296509 | 2.44E-61 |
| F | 462 | 76.407 | 357338 | 357793 | 337266 | 337722 | 2.44E-61 | F | 115 | 100 | 231497 | 231611 | 157768 | 157882 | 1.48E-53 |
| F | 115 | 100 | 231515 | 231629 | 157786 | 157900 | 1.48E-53 | F | 152 | 92.105 | 230544 | 230690 | 136579 | 136730 | 1.91E-52 |
| F | 152 | 92.105 | 230562 | 230708 | 136596 | 136747 | 1.91E-52 | P | 152 | 92.105 | 342216 | 342367 | 230544 | 230690 | 1.91E-52 |
| P | 152 | 92.105 | 255024 | 255175 | 230562 | 230708 | 1.91E-52 | F | 104 | 100 | 339415 | 339518 | 252208 | 252311 | 1.93E-47 |
| F | 104 | 100 | 380587 | 380690 | 252223 | 252326 | 1.93E-47 | P | 151 | 89.404 | 423602 | 423751 | 263552 | 263699 | 8.96E-46 |
| F | 151 | 89.404 | 351937 | 352084 | 272303 | 272452 | 8.96E-46 | P | 151 | 89.404 | 423602 | 423751 | 310724 | 310871 | 8.96E-46 |
| F | 151 | 89.404 | 391931 | 392078 | 272303 | 272452 | 8.96E-46 | P | 124 | 92.742 | 207410 | 207533 | 41780 | 41903 | 1.50E-43 |
| P | 124 | 92.742 | 207428 | 207551 | 41795 | 41918 | 1.50E-43 | P | 95 | 100 | 243318 | 243412 | 231584 | 231678 | 1.94E-42 |
| P | 95 | 100 | 243336 | 243430 | 231602 | 231696 | 1.94E-42 | F | 171 | 85.965 | 233913 | 234075 | 69166 | 69329 | 3.25E-40 |
| F | 171 | 85.965 | 233931 | 234093 | 69182 | 69345 | 3.24E-40 | F | 91 | 100 | 446267 | 446357 | 222919 | 223009 | 3.25E-40 |
| F | 91 | 100 | 446214 | 446304 | 222937 | 223027 | 3.24E-40 | P | 97 | 97.938 | 252769 | 252865 | 160898 | 160993 | 1.17E-39 |
| P | 97 | 97.938 | 381148 | 381244 | 160916 | 161011 | 1.17E-39 | F | 228 | 81.14 | 425766 | 425981 | 100825 | 101041 | 5.43E-38 |
| P | 228 | 81.14 | 270071 | 270286 | 100844 | 101060 | 5.43E-38 | P | 162 | 85.802 | 410027 | 410186 | 135987 | 136137 | 1.95E-37 |
| F | 162 | 85.802 | 285870 | 286029 | 136004 | 136154 | 1.95E-37 | F | 165 | 85.455 | 410024 | 410186 | 342806 | 342959 | 1.95E-37 |
| P | 165 | 85.455 | 285870 | 286032 | 255614 | 255767 | 1.95E-37 | P | 165 | 85.455 | 135987 | 136140 | 410024 | 410186 | 1.95E-37 |
| P | 162 | 85.802 | 255617 | 255767 | 285870 | 286029 | 1.95E-37 | F | 223 | 81.166 | 226316 | 226521 | 54919 | 55133 | 2.53E-36 |
| F | 223 | 81.166 | 226334 | 226539 | 54935 | 55149 | 2.53E-36 | P | 94 | 94.681 | 507774 | 507867 | 1142 | 1233 | 5.47E-33 |
| P | 94 | 94.681 | 507721 | 507814 | 1142 | 1233 | 5.47E-33 | F | 176 | 81.818 | 347692 | 347861 | 215045 | 215219 | 7.07E-32 |
| P | 176 | 81.818 | 435330 | 435499 | 215063 | 215237 | 7.07E-32 | P | 92 | 92.391 | 350709 | 350799 | 60814 | 60905 | 1.53E-28 |
| F | 92 | 92.391 | 432392 | 432482 | 60830 | 60921 | 1.53E-28 | P | 70 | 98.571 | 398171 | 398240 | 320047 | 320116 | 7.13E-27 |
| F | 315 | 75.873 | 351313 | 351623 | 271457 | 271738 | 1.98E-27 | F | 64 | 100 | 491633 | 491696 | 233587 | 233650 | 3.31E-25 |
| F | 315 | 75.873 | 391307 | 391617 | 271457 | 271738 | 1.98E-27 | P | 64 | 100 | 491634 | 491697 | 460278 | 460341 | 3.31E-25 |
| F | 70 | 98.571 | 361218 | 361287 | 297816 | 297885 | 7.12E-27 | P | 63 | 100 | 460279 | 460341 | 233588 | 233650 | 1.19E-24 |
| F | 64 | 100 | 491580 | 491643 | 233605 | 233668 | 3.31E-25 | P | 110 | 86.364 | 424067 | 424173 | 263317 | 263426 | 1.19E-24 |
| P | 64 | 100 | 491581 | 491644 | 460225 | 460288 | 3.31E-25 | P | 110 | 86.364 | 424067 | 424173 | 310489 | 310598 | 1.19E-24 |
| P | 63 | 100 | 460226 | 460288 | 233606 | 233668 | 1.19E-24 | P | 107 | 86.916 | 310492 | 310598 | 424067 | 424170 | 1.19E-24 |
| F | 110 | 86.364 | 351702 | 351811 | 271881 | 271987 | 1.19E-24 | P | 107 | 86.916 | 263320 | 263426 | 424067 | 424170 | 1.19E-24 |
| F | 110 | 86.364 | 391696 | 391805 | 271881 | 271987 | 1.19E-24 | P | 68 | 97.059 | 233588 | 233655 | 79529 | 79596 | 4.29E-24 |
| P | 68 | 97.059 | 233606 | 233673 | 79546 | 79613 | 4.29E-24 | P | 77 | 93.506 | 521727 | 521803 | 231463 | 231539 | 4.29E-24 |
| P | 77 | 93.506 | 521675 | 521751 | 231481 | 231557 | 4.29E-24 | F | 75 | 94.667 | 301601 | 301674 | 295731 | 295804 | 4.29E-24 |
| F | 75 | 94.667 | 342814 | 342887 | 336944 | 337017 | 4.29E-24 | F | 75 | 94.667 | 466976 | 467048 | 334213 | 334286 | 1.54E-23 |
| F | 75 | 94.667 | 466923 | 466995 | 375385 | 375458 | 1.54E-23 | F | 91 | 89.011 | 446034 | 446123 | 96832 | 96922 | 5.55E-23 |
| F | 81 | 92.593 | 358680 | 358755 | 341358 | 341438 | 5.55E-23 | F | 81 | 92.593 | 317509 | 317584 | 300145 | 300225 | 5.55E-23 |
| F | 71 | 94.366 | 446000 | 446070 | 96869 | 96939 | 1.99E-22 | F | 62 | 98.387 | 243784 | 243845 | 121209 | 121270 | 2.00E-22 |
| F | 62 | 98.387 | 243802 | 243863 | 121226 | 121287 | 1.99E-22 | P | 59 | 100 | 297338 | 297396 | 224026 | 224084 | 2.00E-22 |
| P | 59 | 100 | 338551 | 338609 | 224044 | 224102 | 1.99E-22 | F | 64 | 96.875 | 460279 | 460342 | 79534 | 79597 | 7.18E-22 |
| F | 64 | 96.875 | 460226 | 460289 | 79551 | 79614 | 7.17E-22 | P | 58 | 100 | 355874 | 355931 | 260425 | 260482 | 7.18E-22 |
| F | 58 | 100 | 427260 | 427317 | 388804 | 388861 | 7.17E-22 | P | 63 | 96.825 | 491634 | 491696 | 79534 | 79596 | 2.58E-21 |
| P | 63 | 96.825 | 491581 | 491643 | 79551 | 79613 | 2.58E-21 | P | 57 | 100 | 229216 | 229272 | 152927 | 152983 | 2.58E-21 |
| P | 57 | 100 | 229234 | 229290 | 152945 | 153001 | 2.58E-21 | P | 315 | 74.603 | 424316 | 424595 | 262928 | 263238 | 3.34E-20 |
| P | 213 | 76.526 | 366763 | 366964 | 338100 | 338308 | 3.34E-20 | P | 213 | 76.526 | 325592 | 325793 | 296887 | 297095 | 3.34E-20 |
| P | 223 | 75.785 | 338100 | 338320 | 366751 | 366964 | 3.34E-20 | P | 315 | 74.603 | 424316 | 424595 | 310100 | 310410 | 3.34E-20 |
| F | 74 | 91.892 | 245003 | 245071 | 131528 | 131601 | 4.32E-19 | P | 223 | 75.785 | 296887 | 297107 | 325580 | 325793 | 3.34E-20 |
| P | 74 | 91.892 | 260170 | 260243 | 245003 | 245071 | 4.32E-19 | F | 74 | 91.892 | 244985 | 245053 | 131511 | 131584 | 4.32E-19 |
| F | 74 | 91.892 | 435756 | 435829 | 245003 | 245071 | 4.32E-19 | P | 74 | 91.892 | 347362 | 347435 | 244985 | 245053 | 4.32E-19 |
| P | 74 | 90.541 | 446364 | 446437 | 336838 | 336911 | 4.32E-19 | F | 74 | 91.892 | 435809 | 435882 | 244985 | 245053 | 4.32E-19 |
| F | 66 | 93.939 | 388537 | 388601 | 152947 | 153010 | 1.55E-18 | P | 74 | 90.541 | 446417 | 446490 | 295625 | 295698 | 4.32E-19 |
| P | 78 | 89.744 | 47264 | 47336 | 1148 | 1225 | 5.59E-18 | F | 66 | 93.939 | 260158 | 260222 | 152929 | 152992 | 1.55E-18 |
| F | 63 | 93.651 | 207548 | 207610 | 41905 | 41967 | 5.59E-18 | P | 78 | 89.744 | 47249 | 47321 | 1148 | 1225 | 5.59E-18 |
| P | 52 | 98.077 | 388537 | 388588 | 229237 | 229288 | 7.23E-17 | F | 63 | 93.651 | 207530 | 207592 | 41890 | 41952 | 5.59E-18 |
| F | 49 | 100 | 493858 | 493906 | 464497 | 464545 | 7.23E-17 | P | 52 | 98.077 | 260158 | 260209 | 229219 | 229270 | 7.23E-17 |
| F | 54 | 94.444 | 222689 | 222742 | 96882 | 96935 | 1.21E-14 | F | 49 | 100 | 493911 | 493959 | 464550 | 464598 | 7.23E-17 |
| F | 48 | 97.917 | 340526 | 340573 | 131490 | 131537 | 1.21E-14 | F | 47 | 100 | 201326 | 201372 | 158312 | 158358 | 9.35E-16 |
| P | 48 | 97.917 | 340526 | 340573 | 260234 | 260281 | 1.21E-14 | F | 46 | 100 | 278942 | 278987 | 278909 | 278954 | 3.36E-15 |
| F | 48 | 97.917 | 435718 | 435765 | 340526 | 340573 | 1.21E-14 | F | 54 | 94.444 | 222671 | 222724 | 96865 | 96918 | 1.21E-14 |
| F | 93 | 83.871 | 507715 | 507807 | 47252 | 47336 | 4.35E-14 | F | 48 | 97.917 | 299313 | 299360 | 131473 | 131520 | 1.21E-14 |
| F | 44 | 100 | 388549 | 388592 | 374787 | 374830 | 4.35E-14 | P | 48 | 97.917 | 347426 | 347473 | 299313 | 299360 | 1.21E-14 |
| P | 61 | 90.164 | 53310 | 53369 | 26716 | 26776 | 5.63E-13 | F | 48 | 97.917 | 435771 | 435818 | 299313 | 299360 | 1.21E-14 |
| P | 58 | 91.379 | 26719 | 26776 | 53310 | 53366 | 5.63E-13 | F | 93 | 83.871 | 507768 | 507860 | 47237 | 47321 | 4.35E-14 |
| F | 60 | 90 | 207374 | 207432 | 41730 | 41789 | 2.02E-12 | F | 44 | 100 | 333615 | 333658 | 260170 | 260213 | 4.35E-14 |
| F | 40 | 100 | 285763 | 285802 | 57421 | 57460 | 7.28E-12 | P | 61 | 90.164 | 53294 | 53353 | 26712 | 26772 | 5.63E-13 |
| F | 40 | 100 | 407276 | 407315 | 303077 | 303116 | 7.28E-12 | P | 58 | 91.379 | 26715 | 26772 | 53294 | 53350 | 5.63E-13 |
| F | 42 | 97.619 | 179275 | 179316 | 53099 | 53140 | 2.62E-11 | F | 60 | 90 | 207356 | 207414 | 41715 | 41774 | 2.02E-12 |
| P | 57 | 89.474 | 294269 | 294325 | 175128 | 175183 | 9.41E-11 | P | 40 | 100 | 410254 | 410293 | 57405 | 57444 | 7.28E-12 |
| F | 40 | 97.5 | 374787 | 374826 | 152959 | 152998 | 3.39E-10 | P | 40 | 100 | 392940 | 392979 | 278907 | 278946 | 7.28E-12 |
| P | 40 | 97.5 | 374787 | 374826 | 229237 | 229276 | 3.39E-10 | F | 42 | 97.619 | 179257 | 179298 | 53083 | 53124 | 2.62E-11 |
| F | 39 | 97.436 | 464881 | 464919 | 182446 | 182484 | 1.22E-09 | F | 57 | 89.474 | 401731 | 401787 | 175110 | 175165 | 9.41E-11 |
| F | 36 | 100 | 499794 | 499829 | 499522 | 499557 | 1.22E-09 | P | 45 | 95.556 | 392940 | 392984 | 278936 | 278979 | 9.41E-11 |
| F | 48 | 91.667 | 312419 | 312465 | 119612 | 119658 | 4.38E-09 | F | 40 | 97.5 | 333615 | 333654 | 152941 | 152980 | 3.39E-10 |
| P | 41 | 95.122 | 214851 | 214891 | 153459 | 153499 | 4.38E-09 | P | 40 | 97.5 | 333615 | 333654 | 229219 | 229258 | 3.39E-10 |
| F | 47 | 91.489 | 201344 | 201390 | 158330 | 158376 | 4.38E-09 | F | 39 | 97.436 | 464934 | 464972 | 182428 | 182466 | 1.22E-09 |
| P | 38 | 97.368 | 153462 | 153499 | 214851 | 214888 | 4.38E-09 | F | 36 | 100 | 499847 | 499882 | 499575 | 499610 | 1.22E-09 |
| P | 34 | 100 | 507545 | 507578 | 49093 | 49126 | 1.58E-08 | P | 48 | 91.667 | 383591 | 383637 | 119595 | 119641 | 4.38E-09 |
| F | 37 | 97.297 | 351723 | 351759 | 112498 | 112534 | 1.58E-08 | P | 41 | 95.122 | 214833 | 214873 | 153441 | 153481 | 4.38E-09 |
| F | 37 | 97.297 | 391717 | 391753 | 112498 | 112534 | 1.58E-08 | P | 38 | 97.368 | 153444 | 153481 | 214833 | 214870 | 4.38E-09 |
| F | 44 | 93.182 | 508352 | 508393 | 203566 | 203609 | 1.58E-08 | P | 45 | 93.333 | 119598 | 119641 | 383591 | 383634 | 4.38E-09 |
| P | 45 | 91.111 | 297447 | 297491 | 145172 | 145216 | 5.66E-08 | F | 35 | 100 | 456802 | 456836 | 432076 | 432110 | 4.38E-09 |
| P | 33 | 100 | 456751 | 456783 | 263942 | 263974 | 5.66E-08 | P | 34 | 100 | 507598 | 507631 | 49078 | 49111 | 1.58E-08 |
| F | 61 | 85.246 | 351827 | 351887 | 272189 | 272247 | 5.66E-08 | F | 40 | 95 | 364300 | 364339 | 103496 | 103535 | 1.58E-08 |
| F | 61 | 85.246 | 391821 | 391881 | 272189 | 272247 | 5.66E-08 | F | 37 | 97.297 | 263338 | 263374 | 112481 | 112517 | 1.58E-08 |
| P | 45 | 91.111 | 508440 | 508484 | 435532 | 435576 | 5.66E-08 | F | 37 | 97.297 | 310510 | 310546 | 112481 | 112517 | 1.58E-08 |
| F | 38 | 94.737 | 353264 | 353301 | 83678 | 83715 | 2.04E-07 | F | 44 | 93.182 | 508405 | 508446 | 203548 | 203591 | 1.58E-08 |
| P | 32 | 100 | 243306 | 243337 | 243306 | 243337 | 2.04E-07 | F | 45 | 91.111 | 398565 | 398609 | 145154 | 145198 | 5.67E-08 |
| F | 35 | 97.143 | 351631 | 351665 | 271767 | 271801 | 2.04E-07 | P | 61 | 85.246 | 423807 | 423865 | 263442 | 263502 | 5.67E-08 |
| F | 35 | 97.143 | 391625 | 391659 | 271767 | 271801 | 2.04E-07 | P | 61 | 85.246 | 423807 | 423865 | 310614 | 310674 | 5.67E-08 |
| P | 42 | 92.857 | 356312 | 356353 | 291423 | 291462 | 2.04E-07 | F | 45 | 91.111 | 508493 | 508537 | 347615 | 347659 | 5.67E-08 |
| F | 35 | 97.143 | 389394 | 389428 | 295805 | 295839 | 2.04E-07 | P | 64 | 84.375 | 310614 | 310677 | 423804 | 423865 | 5.67E-08 |
| P | 40 | 92.5 | 418852 | 418891 | 103515 | 103554 | 7.33E-07 | P | 64 | 84.375 | 263442 | 263505 | 423804 | 423865 | 5.67E-08 |
| F | 34 | 97.059 | 510901 | 510934 | 153467 | 153500 | 7.33E-07 | F | 38 | 94.737 | 312051 | 312088 | 83661 | 83698 | 2.04E-07 |
| P | 43 | 90.698 | 521675 | 521717 | 157786 | 157828 | 7.33E-07 | P | 32 | 100 | 243288 | 243319 | 243288 | 243319 | 2.04E-07 |
| P | 34 | 97.059 | 262837 | 262870 | 169004 | 169037 | 7.33E-07 | P | 35 | 97.143 | 400217 | 400251 | 261015 | 261049 | 2.04E-07 |
| P | 41 | 92.683 | 385244 | 385284 | 182102 | 182140 | 7.33E-07 | P | 35 | 97.143 | 424253 | 424287 | 263246 | 263280 | 2.04E-07 |
| P | 34 | 97.059 | 103521 | 103554 | 418852 | 418885 | 7.33E-07 | P | 35 | 97.143 | 424253 | 424287 | 310418 | 310452 | 2.04E-07 |
| F | 33 | 96.97 | 402413 | 402445 | 94776 | 94808 | 2.64E-06 | F | 42 | 92.857 | 404594 | 404633 | 315101 | 315142 | 2.04E-07 |
| P | 30 | 100 | 426226 | 426255 | 130422 | 130451 | 2.64E-06 | P | 41 | 92.683 | 310418 | 310458 | 424247 | 424287 | 2.04E-07 |
| F | 94 | 78.723 | 137120 | 137208 | 132145 | 132235 | 2.64E-06 | P | 41 | 92.683 | 263246 | 263286 | 424247 | 424287 | 2.04E-07 |
| P | 94 | 78.723 | 259536 | 259626 | 137120 | 137208 | 2.64E-06 | F | 34 | 97.059 | 510954 | 510987 | 153449 | 153482 | 7.33E-07 |
| F | 94 | 78.723 | 436373 | 436463 | 137120 | 137208 | 2.64E-06 | P | 43 | 90.698 | 521727 | 521769 | 157768 | 157810 | 7.33E-07 |
| F | 61 | 83.607 | 242582 | 242641 | 167118 | 167177 | 2.64E-06 | F | 34 | 97.059 | 433182 | 433215 | 168986 | 169019 | 7.33E-07 |
| P | 33 | 96.97 | 235755 | 235787 | 218921 | 218953 | 2.64E-06 | P | 41 | 92.683 | 256865 | 256905 | 182084 | 182122 | 7.33E-07 |
| F | 30 | 100 | 463502 | 463531 | 224140 | 224169 | 2.64E-06 | F | 33 | 96.97 | 274034 | 274066 | 94759 | 94791 | 2.64E-06 |
| P | 30 | 100 | 218924 | 218953 | 235755 | 235784 | 2.64E-06 | F | 30 | 100 | 356936 | 356965 | 130405 | 130434 | 2.64E-06 |
| P | 78 | 80.769 | 137136 | 137208 | 259536 | 259612 | 2.64E-06 | F | 94 | 78.723 | 137103 | 137191 | 132128 | 132218 | 2.64E-06 |
| P | 64 | 82.812 | 90480 | 90541 | 5109 | 5171 | 9.48E-06 | P | 94 | 78.723 | 346728 | 346818 | 137103 | 137191 | 2.64E-06 |
| F | 38 | 92.105 | 72296 | 72333 | 58161 | 58198 | 9.48E-06 | F | 94 | 78.723 | 436426 | 436516 | 137103 | 137191 | 2.64E-06 |
| F | 29 | 100 | 336989 | 337017 | 85490 | 85518 | 9.48E-06 | F | 61 | 83.607 | 242564 | 242623 | 167100 | 167159 | 2.64E-06 |
| F | 29 | 100 | 342859 | 342887 | 85490 | 85518 | 9.48E-06 | P | 33 | 96.97 | 235737 | 235769 | 218903 | 218935 | 2.64E-06 |
| P | 76 | 80.263 | 5109 | 5183 | 90468 | 90541 | 9.48E-06 | F | 30 | 100 | 463555 | 463584 | 224122 | 224151 | 2.64E-06 |
| P | 29 | 100 | 243472 | 243500 | 171368 | 171396 | 9.48E-06 | P | 30 | 100 | 218906 | 218935 | 235737 | 235766 | 2.64E-06 |
| F | 36 | 94.444 | 454397 | 454432 | 238278 | 238312 | 9.48E-06 | P | 78 | 80.769 | 137119 | 137191 | 346728 | 346804 | 2.64E-06 |
| P | 50 | 86 | 422245 | 422294 | 242582 | 242631 | 9.48E-06 | P | 64 | 82.812 | 90463 | 90524 | 5105 | 5167 | 9.48E-06 |
| F | 32 | 96.875 | 517567 | 517598 | 498703 | 498734 | 9.48E-06 | F | 38 | 92.105 | 72280 | 72317 | 58145 | 58182 | 9.48E-06 |
|  |  |  |  |  |  |  |  | F | 29 | 100 | 295776 | 295804 | 85473 | 85501 | 9.48E-06 |
|  |  |  |  |  |  |  |  | F | 29 | 100 | 301646 | 301674 | 85473 | 85501 | 9.48E-06 |
|  |  |  |  |  |  |  |  | P | 76 | 80.263 | 5105 | 5179 | 90451 | 90524 | 9.48E-06 |
|  |  |  |  |  |  |  |  | P | 29 | 100 | 243454 | 243482 | 171350 | 171378 | 9.48E-06 |
|  |  |  |  |  |  |  |  | F | 36 | 94.444 | 454450 | 454485 | 238260 | 238294 | 9.48E-06 |
|  |  |  |  |  |  |  |  | F | 50 | 86 | 360897 | 360946 | 242564 | 242613 | 9.48E-06 |
|  |  |  |  |  |  |  |  | F | 32 | 96.875 | 517619 | 517650 | 498756 | 498787 | 9.48E-06 |

**TableS8A The statistics of homologous fragments between mitochondria and chloroplasts in DF**

| percentage of identical matches | length | number of mismatches | number of gap openings | start of alignment in query | end of alignment in query | start of alignment in subject | end of alignment in subject | expect value | bitscore |
| --- | --- | --- | --- | --- | --- | --- | --- | --- | --- |
| 95.219 | 1527 | 61 | 11 | 106958 | 108479 | 357920 | 356401 | 0 | 2405 |
| 95.219 | 1527 | 61 | 11 | 130771 | 132292 | 356401 | 357920 | 0 | 2405 |
| 95.238 | 1197 | 37 | 17 | 148259 | 149435 | 320742 | 319546 | 0 | 1877 |
| 95.238 | 1197 | 37 | 17 | 89815 | 90991 | 319546 | 320742 | 0 | 1877 |
| 92.716 | 961 | 40 | 21 | 134900 | 135831 | 349002 | 348043 | 0 | 1360 |
| 92.716 | 961 | 40 | 21 | 103419 | 104350 | 348043 | 349002 | 0 | 1360 |
| 85.079 | 1012 | 102 | 18 | 80699 | 81671 | 107848 | 108849 | 0 | 987 |
| 97.308 | 260 | 4 | 3 | 132517 | 132773 | 367010 | 366751 | 6.67E-122 | 438 |
| 97.308 | 260 | 4 | 3 | 106477 | 106733 | 366751 | 367010 | 6.67E-122 | 438 |
| 74.074 | 891 | 173 | 43 | 137262 | 138125 | 223906 | 223047 | 4.22E-84 | 313 |
| 74.074 | 891 | 173 | 43 | 101125 | 101988 | 223047 | 223906 | 4.22E-84 | 313 |
| 91.584 | 202 | 13 | 4 | 100548 | 100745 | 13986 | 13785 | 5.53E-73 | 276 |
| 91.584 | 202 | 13 | 4 | 138505 | 138702 | 13785 | 13986 | 5.53E-73 | 276 |
| 84.404 | 218 | 29 | 3 | 80296 | 80510 | 107594 | 107809 | 5.69E-53 | 209 |
| 87.222 | 180 | 11 | 4 | 66368 | 66541 | 319354 | 319181 | 1.59E-48 | 195 |
| 83.077 | 195 | 28 | 1 | 66702 | 66891 | 319052 | 318858 | 7.47E-42 | 172 |
| 97.531 | 81 | 2 | 0 | 84314 | 84394 | 166377 | 166297 | 7.57E-32 | 139 |
| 97.531 | 81 | 2 | 0 | 154856 | 154936 | 166297 | 166377 | 7.57E-32 | 139 |
| 97.531 | 81 | 2 | 0 | 109103 | 109183 | 232570 | 232490 | 7.57E-32 | 139 |
| 97.531 | 81 | 2 | 0 | 130067 | 130147 | 232490 | 232570 | 7.57E-32 | 139 |
| 98.571 | 70 | 1 | 0 | 106202 | 106271 | 274592 | 274523 | 2.12E-27 | 124 |
| 98.571 | 70 | 1 | 0 | 132979 | 133048 | 274523 | 274592 | 2.12E-27 | 124 |
| 92.208 | 77 | 6 | 0 | 52801 | 52877 | 220205 | 220281 | 5.94E-23 | 110 |
| 91.045 | 67 | 5 | 1 | 105276 | 105341 | 339979 | 339913 | 7.74E-17 | 89.8 |
| 91.045 | 67 | 5 | 1 | 133909 | 133974 | 339913 | 339979 | 7.74E-17 | 89.8 |

**TableS8B The statistics of homologous fragments between mitochondria and chloroplasts in SY**

| percentage of identical matches | length | number of mismatches | number of gap openings | start of alignment in query | end of alignment in query | start of alignment in subject | end of alignment in subject | expect value | bitscore |
| --- | --- | --- | --- | --- | --- | --- | --- | --- | --- |
| 97.727 | 1584 | 28 | 8 | 106860 | 108436 | 316749 | 315167 | 0 | 97.727 |
| 97.727 | 1584 | 28 | 8 | 130615 | 132191 | 315167 | 316749 | 0 | 97.727 |
| 94.904 | 1197 | 41 | 17 | 89716 | 90892 | 376522 | 375326 | 0 | 94.904 |
| 94.904 | 1197 | 41 | 17 | 148159 | 149335 | 375326 | 376522 | 0 | 94.904 |
| 92.716 | 961 | 40 | 21 | 134799 | 135730 | 307789 | 306830 | 0 | 92.716 |
| 92.716 | 961 | 40 | 21 | 103321 | 104252 | 306830 | 307789 | 0 | 92.716 |
| 85.997 | 607 | 68 | 11 | 80601 | 81199 | 107829 | 108426 | 0 | 85.997 |
| 88.391 | 379 | 35 | 5 | 81203 | 81573 | 108455 | 108832 | 1.11E-124 | 88.391 |
| 97.308 | 260 | 4 | 3 | 132416 | 132672 | 325839 | 325580 | 6.67E-122 | 97.308 |
| 97.308 | 260 | 4 | 3 | 106379 | 106635 | 325580 | 325839 | 6.67E-122 | 97.308 |
| 74.074 | 891 | 173 | 43 | 137162 | 138025 | 223888 | 223029 | 4.22E-84 | 74.074 |
| 74.074 | 891 | 173 | 43 | 101026 | 101889 | 223029 | 223888 | 4.22E-84 | 74.074 |
| 91.584 | 202 | 13 | 4 | 100449 | 100646 | 13982 | 13781 | 5.53E-73 | 91.584 |
| 91.584 | 202 | 13 | 4 | 138405 | 138602 | 13781 | 13982 | 5.53E-73 | 91.584 |
| 84.404 | 218 | 29 | 3 | 80198 | 80412 | 107575 | 107790 | 5.69E-53 | 84.404 |
| 87.222 | 180 | 11 | 4 | 66312 | 66485 | 376714 | 376887 | 1.59E-48 | 87.222 |
| 82.234 | 197 | 28 | 3 | 66646 | 66837 | 377016 | 377210 | 4.49E-39 | 82.234 |
| 97.531 | 81 | 2 | 0 | 84215 | 84295 | 166359 | 166279 | 7.57E-32 | 97.531 |
| 97.531 | 81 | 2 | 0 | 154756 | 154836 | 166279 | 166359 | 7.57E-32 | 97.531 |
| 97.531 | 81 | 2 | 0 | 109005 | 109085 | 232552 | 232472 | 7.57E-32 | 97.531 |
| 97.531 | 81 | 2 | 0 | 129966 | 130046 | 232472 | 232552 | 7.57E-32 | 97.531 |
| 98.571 | 70 | 1 | 0 | 132878 | 132947 | 421531 | 421462 | 2.12E-27 | 98.571 |
| 98.571 | 70 | 1 | 0 | 106104 | 106173 | 421462 | 421531 | 2.12E-27 | 98.571 |
| 92.208 | 77 | 6 | 0 | 52774 | 52850 | 220187 | 220263 | 5.94E-23 | 92.208 |
| 91.045 | 67 | 5 | 1 | 105178 | 105243 | 298766 | 298700 | 7.73E-17 | 91.045 |
| 91.045 | 67 | 5 | 1 | 133808 | 133873 | 298700 | 298766 | 7.73E-17 | 91.045 |

**TableS9 The Genbank number used in phylogenetic tree**

| Species | Genbank(CP) | Genbank(MT) | Family |
| --- | --- | --- | --- |
| *Polygonatum kingianum* | OR995658 | PP861176 | Asparagaceae |
| *Eucalyptus rudis* | PP920097 | PP920092 | Myrtaceae |
| *Dracaena sp.* | OR601564 | OQ445559 | Asparagaceae |
| *Dracaena cochinchinensis* | MN200195 | PP704687 | Asparagaceae |
| *Crocus sativus* | OM772765 | OL804177 | Iridaceae |
| *Chlorophytum comosum* | MN871944 | MW411187 | Asparagaceae |
| *Asparagus officinalis* | ON872702 | NC_053642 | Asparagaceae |
| *Asparagus officinalis* | NC_034777 | MT483944 | Asparagaceae |
| *Asparagus officinalis* | KY364194 |  | Asparagaceae |
| *Apostasia shenzhenica* | NC_039812 | OQ645347 | Orchidaceae |
| *Apostasia shenzhenica* | MG772639 | NC_077647 | Orchidaceae |
| *Apostasia fujianica* | PP599181 | PP724664 | Orchidaceae |
| *Allium fistulosum* | NC_040222 | OL347690 | Amaryllidaceae |
| *Allium cepa* | OR783242 | OQ473923 | Amaryllidaceae |
| *Allium cepa* | OQ473924 | NC_030100 | Amaryllidaceae |
